# Two replications of Raymond, Shapiro, and Arnell (1992), The Attentional Blink

**DOI:** 10.3758/s13428-020-01457-6

**Published:** 2020-08-12

**Authors:** Massimo Grassi, Camilla Crotti, David Giofrè, Ingrid Boedker, Enrico Toffalini

**Affiliations:** 1 Department of General Psychology, University of Padova, Via Venezia 8, 35131 Padova, Italy; 2 DISFOR, University of Genoa, Genoa, Italy

**Keywords:** Attentional blink, Replication

## Abstract

In order to improve the trustworthiness of our science, several new research practices have been suggested, including preregistration, large statistical power, availability of research data and materials, new statistical standards, and the replication of experiments. We conducted a replication project on an original phenomenon that was discovered more than 25 years ago, namely the attentional blink (Raymond, Shapiro, & Arnell, Human Perception and Performance, 18(3), 849–860, 1992), which has been conceptually replicated hundreds of times with major variations. Here, we ran two identical experiments, adopting the new practices and closely reproducing the original experiment. The two experiments were run by different research groups in different countries and laboratories with different participants. Experiment 1 shared remarkable similarities (in magnitude and duration of the effect) with the original study, but also some differences (the overall accuracy of participants, the timing of the effect, and lag-1 sparing). Experts interviewed to evaluate our results stressed the similarities rather than the differences. Experiment 2 replicated nearly identically the results observed in Experiment 1. These findings show that the adoption of new research practices improves the replicability of experimental research and opens the door for a quantitative and direct comparison of the results collected across different laboratories and countries.

**Electronic supplementary material:**

The online version of this article (10.3758/s13428-020-01457-6) contains supplementary material, which is available to authorized users.

Replicability is an essential feature of scientific research. Recently, several new research practices have been suggested to improve replicability. These include preregistration (e.g., Lindsay, Simons, & Lilienfeld, 2016), increasing statistical power (e.g., by using larger samples, Tressoldi & Giofrè, 2015; Wagenmakers et al., 2014), and making data and digital materials available (Bakker & Wicherts, 2014; Wicherts & Bakker, 2012). All these practices are currently being encouraged by major journals and societies (e.g., Cowan, 2018). New practices also extend to the interpretation of the results, where the focus on the effect size and its uncertainty—rather than the mere statistical significance—is a crucial practice which is not always observed (Bakker & Wicherts, 2011; Cumming et al., 2007; Giofrè et al., 2017), despite having been explicitly recommended by the American Psychological Association (APA) since 2001 (APA Publication manual, 2010). Emphasizing the quantification of the effect size with precision implies spending one’s efforts on the accurate description of an effect from a quantitative point of view, rather than limiting one’s reasoning to simplified dichotomous decisions between rejection and non-rejection of null hypotheses (e.g., see Kruschke & Liddell, 2015).

Empirical replication is the main tool to assess the replicability of a study and its findings. In psychology, it is not possible to implement the exact replication of a study (see Zwaan, et al., 2018, for an extensive discussion). Even if we run the same experiment twice, with the same participants, they will not be in the exact mental state they were in during the first experiment. Replications are divided into two types: direct replications and conceptual replications (Zwaan, Etz, Lucas, & Donnellan, 2018; Chambers, 2017). Direct replications attempt to replicate all the theoretically relevant elements of an original study given the current understanding of the phenomenon. For example, if we are replicating a digit span experiment that presents Arabic digits, we should not use Roman numerals for the replication: familiarity has been shown to affect our ability to remember stimuli (e.g., Hulme, Maughan, & Brown, 1991). We can assume, by contrast, that presenting the same Arabic digits in different fonts (e.g., Arial versus Times New Roman) may not be theoretically relevant, given our current understanding of memory. Conceptual replications, on the other hand, investigate an extension of a given finding or paradigm. For example, an experimenter may run a study based on a given experimental paradigm (e.g., digit span) but changing the stimuli of the original paradigm, for example, from visual to auditory ones (Zwaan, et al., 2018). For the purpose of falsification (e.g., when we assess whether or not a given phenomenon exists), the epistemological value of direct replications is greater than that of conceptual replications (Pashler & Harris, 2012). In the case of truly existing effects, replications (either direct or conceptual) enable the precise assessment of a phenomenon’s characteristics. For example, direct replications enable one to assess the variability related to the sampling of participants, a major source of variability when conducting a replication (Klein et al., 2018). This is particularly interesting when effects are small and difficult to capture. Conceptual replications, in contrast, elucidate how a given change in the experimental paradigm changes the picture of the phenomenon.

In this study, we replicated Experiment 2 from Raymond, Shapiro, and Arnell’s (1992) seminal paper that made the attentional blink phenomenon famous (the phenomenon was first revealed by Broadbent & Broadbent, 1987, and Weichselgartner & Sperling, 1987). This classic paper, highlighting the temporal limits of human attention when processing a stimulus in a rapid serial visual presentation (RSVP), has had a tremendous influence on psychological research: the original paper has been cited over 2800 times (source: Google Scholar, June 2020), and the “blink” has been observed in hundreds of successive studies (see Dux & Marois, 2009; MacLean & Arnell, 2012; Martens, & Wyble, 2010 for a review). The results of that experiment became iconic: the graph representing the original results has been reproduced in a number of successive papers (e.g., Shapiro, 1994; Shapiro, Raymond, & Arnell, 1997b) and it is still a benchmark for the outcome of this classic paradigm^1^. The present study attempted to replicate as closely as possible the characteristics of Experiment 2.

In the original experiment, participants were presented with a fast stream of letters displayed at the center of a gray background. Letters were black except for one that was white (called the target). In half of the trials, the stream included the letter “X” (called the probe), while in the other half of the trials, it did not. The probe could appear in nine positions. It could appear in the target position or “lag” up to eight “relative serial positions” after the target. In the control condition, the participant was asked to detect the probe (i.e., the letter “X”), and detection was almost at ceiling (M = 91%) regardless of the lag. In the experimental condition, the participant first had to report the identity of the target and then indicate the presence or absence of the probe (see Raymond et al. 1992, Fig. 1). Probe detection started at 100% when the probe was presented at target position (i.e., lag 0), dropped linearly just below 50% when the probe was at lag 3, and then increased linearly at 100% when the probe was at lag 8. Because in the control condition probe detection was almost at ceiling (i.e., ~90%) and independent of the lag (two results observed in a number of successive experiments using control-like conditions, see Raymond et al., 1992, Experiment 3; Shapiro et al. 1994, Experiments 1, 2, 3A, 3B, 4, 5A, 5B; Raymond et al. 1995, Experiment 1), the drop in performance observed in the experimental condition could not be regarded as a sensory/perceptual phenomenon. The blink, in fact, was observed in two previous studies (i.e., Broadbent & Broadbent, 1987; Weichselgartner & Sperling, 1987), but these studies used rather complex stimuli or tasks and could not disentangle the attentional/sensory issue. Raymond et al. (1992) had the advantage of simplifying stimulus and task and introducing the control condition that enabled them to clearly reveal the attentional nature of the phenomenon. The experiment was interpreted as revealing the temporal limits of attention that needs time to recover from the processing of the target before processing the probe (this attentional interpretation has been challenged, however: Raymond and colleagues’ blink could also arise from a task-switching cost (e.g., McLaughlin, Shore, & Klein, 2001). Another noticeable result of the experiment was the so-called lag-1 sparing: the worst performance in the experimental condition was not observed when the probe was presented immediately after the target. Surprisingly, at lag 1, the detection performance was still high, at about 80% (i.e., “lag-1 sparing”).

**Fig. 1:**
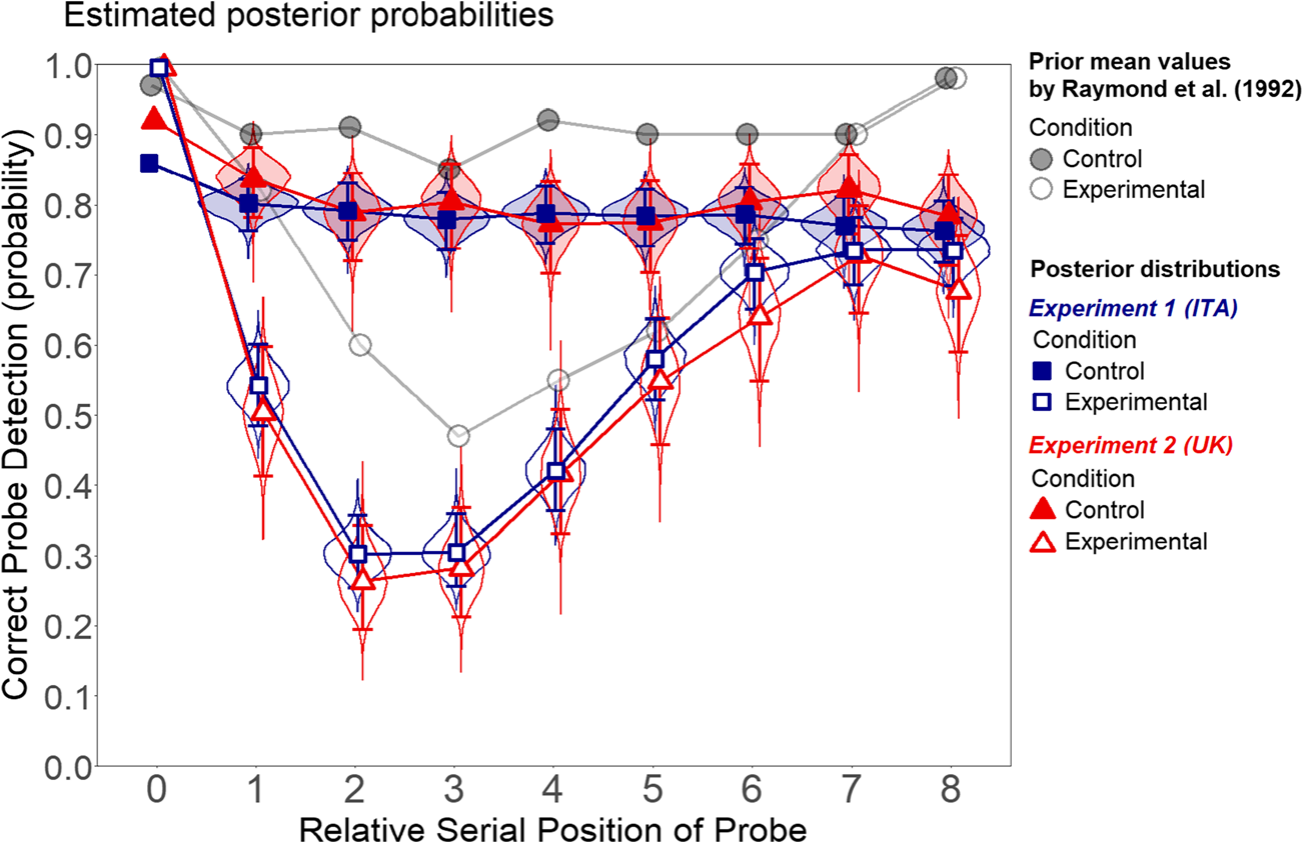
Estimated accuracy of correct probe detection (conditional on target identification) as a function of Condition and Lag in Experiments 1 and 2. Lag is here referred to as “relative serial position of probe” like in the original experiment by Raymond et al. (1992). The error bars represent the 95% Bayesian credible intervals. The violins represent the entire posterior distributions. The circles represent the estimated prior mean values (thus reproducing the graph in the top panel of Fig. 3 in Raymond et al., 1992). Lag 0 was not included in the model, and its mean accuracies are shown for descriptive purposes only

The current study is divided into two experiments. Experiment 1 is a replication of the original study by Raymond et al. (1992) that has been rerun with a sample size ten times as large as the original experiment to obtain an accurate account of the phenomenon, with minimal uncertainty on the estimated parameters. Like many classic experiments, the original study on the attentional blink was conducted with a limited number of participants (*N* = 10). In addition, although the original study collected dichotomous responses (“probe absent/probe present”) that are not normally distributed—especially when performance is close to or at ceiling, as in the original findings—the authors run statistical tests that assume a normal distribution (i.e., analysis of variance [ANOVA]). Here, along with the original analysis, an appropriate statistical analysis was performed (i.e., logistic regression to model binomial responses). The comparison of original results with the outcome of Experiment 1 was performed both statistically and subjectively. We asked experts in attentional blink whether they thought that our results replicated those of the original study.

Experiment 1 set the path for Experiment 2, which was a direct replication of Experiment 1 performed by an independent research team, using the same materials. In both experiments, a Bayesian approach to data analysis was adopted because of the following advantages (Kruschke, 2014; van de Schoot et al., 2014): (i) the ability to include prior evidence (i.e., the results of Raymond et al., 1992) in the data analysis to precisely quantify whether and how newly collected data would deviate from the available evidence; and (ii) the emphasis on the estimation of effect uncertainty (Kruschke & Liddell, 2015), rather than the simplified reject/not-reject dichotomous choice of the traditional null hypothesis significance testing (NHST; McElreath, 2016). Because of the multifaceted nature of the attentional blink (typical of many experiments in experimental psychology), we concentrated on several effect sizes. We sought to quantify all different aspects of this phenomenon (by reporting posterior model coefficients and estimates), in order to assess its exact nature and describe as best as possible whether and how it differed from the original findings (i.e., by Raymond et al., 1992). Both Experiment 1 and Experiment 2 were preregistered (https://osf.io/hp9nk/).

Altogether, this double replication should reveal the degree to which the attentional blink phenomenon can be described with precision, using the original findings of Raymond et al. (1992), as a first term for comparison. Independently repeating the same experiment twice will enable us to assess the internal validity of replication, that is, how much the adoption of the abovementioned practices (preregistration, large statistical power, availability of research data and materials, new statistical standards) enables one to replicate with precision the findings of a multifactorial, complex phenomenon such as the attentional blink.

## Experiment 1

### Method

#### Participants

As in the original study, university students (University of Padova) volunteered to participate and were naive to the purpose of the study. They did not receive any payment or course credit. Participants were 100 undergraduates (mean age = 21.25, SD = 3.47, range: 18 to 46 years, females = 70). Gender distribution was identical to Experiment 2 of Raymond et al. (1992). As described in the preregistration, the estimation of the sample size was based on an extremely conservative hypothesis on the effect size of interest. The latter was defined as the maximum difference in detection probe accuracy between the control and experimental conditions. An extremely conservative lower bound for the effect of interest was Cohen’s *d* = .30, i.e., what could be defined as a “small” effect. With α = .05, power = .90, paired observations, and a one-sided test (because Raymond et al., 1992, provided information on the direction of the effects), the estimated *N* was 97, which we rounded up to 100. We chose a sample size much larger than that of Raymond et al. (1992), based on a conservative power analysis to reduce the uncertainty as much as possible, i.e., to describe the phenomenon in as much detail as possible, under the conditions of our study.

#### Apparatus, stimuli, and procedure

The study was approved by the local ethics committee. Stimuli were generated via an Asus T3 computer, displayed on an NEC MultiSync P1150 17-inch color CRT monitor. The refresh rate of the original monitor was not reported in the original study (Raymond et al. 1992), but stimulus duration was compatible with a refresh rate of 66 Hz^2^ (i.e., frame duration 15.15 ms). Therefore, we ran half of the participants (35 females) with a 60 Hz refresh rate (i.e., frame duration 16.66 ms) and half of the participants (35 females) with a 70 Hz refresh rate (i.e., frame duration 14.28 ms) to target a “virtual” average refresh rate of 65 Hz (see below). As in Raymond et al. (1992), the participants viewed the stimulus binocularly from a distance of 35 cm and stabilized their head position with the aid of a chin rest. The experimenter, who collected and typed in the responses for the participants, sat behind the monitor and could not see the stimulus^3^. Luminance (i.e., white 32.9 cd/m^2^; gray 9.1 cd/m^2^; black, not reported in the original paper, [0, 0, 0] RGB) and physical and retinal size (i.e., letter height 0.82°) of the stimuli were calibrated to be identical to those reported in the original study.

- ***Stimulus.*** The stimulus consisted of a stream of black capital letters presented individually in rapid succession, one after another, at the center of a gray background. Each letter was printed on the screen for one frame only and was separated from successive letters by a blank, fixed duration interval of five frames. A custom software program generated a new stream for each trial. The stream was a random permutation of the 26 letters of the English alphabet, excluding the letter “X.” The stream included one white letter called “target” that was randomly selected in each trial. The number of pre-target letters was random and varied between 7 and 15. In the experiment, in half of the trials, the “X” letter (i.e., the probe) was added to the stream. The probe could be white in target position (i.e., lag 0) or black (i.e., like the other letters of the stream) and lag up to eight letters after the target (respectively, lag 1 to lag 8) totaling nine different probe positions. The probe was followed by a minimum of two letters (when the target was in position 16 and probe in lag 8) up to a maximum of 18 letters (when the target and probe coincided and were in position 8). The stream was preceded by a fixation point (i.e., “*”, 12 frames) that was followed by a blank interval (five frames).
- ***Procedure.*** The experiment included two conditions counterbalanced within each group of participants (i.e., the 60 Hz frame rate group and the 70 Hz frame rate group): the control condition and the experimental condition. In both, the participant sat 35 cm from the screen, with the distance between the participant’s eyes and screen kept fixed by means of a chin rest. In both conditions, the computer ran 180 trials. In the control condition, the participant was asked to look at the stream and to report to the experimenter whether or not the stream included the probe. In the experimental condition, the participant was asked to communicate to the experimenter the identity of the target and successively whether or not the stream included the probe. In both conditions, the probe was presented randomly in half of the trials. The control and the experimental conditions were preceded by six familiarization trials. The participant could take breaks at any time during the experiment.

#### Differences in the stimuli and laboratory conditions

Here we list the differences we are aware of between the current replication and the original experiment.

- ***Differences in the timing of the stimulus.*** Because of the difference in frame duration, there were subtle differences in the timing of the stimuli. The frame duration determines the exact stimulus onset asynchrony (SOA) of the letters of the stream. The rate of stimulus presentation is a theoretically relevant factor because it is known to modulate the width of the blink monotonically (i.e., the shorter the SOA, the larger the width: see Popple & Levi, 2007). In the original paper, each letter remained on the screen for 15.15 ms followed by a 75.75 ms blank interval (SOA = 90.09 ms). In our experiment, each letter remained on the screen for 14.28 ms (or 16.66 ms) followed by a 71.4 ms (or 83.3 ms) blank. Therefore, SOAs were, respectively, 85.71 ms and 100 ms. Because of the different frame duration, the exact duration of the fixation dot before the stimulus was also different: 180 ms in the original study, 171.36 ms (or 199.92 ms) in our experiment.
- ***Differences in the font of the stimulus.*** Another possible difference between the current direct replication and the original study is the font used for the letters of the stream. Raymond et al. (1992) wrote that the font of the letters was “block style, upper case.” The Fig. 1 included in the paper (see also Fig. 1 of Shapiro et al., 1994, and Fig. 1 of Shapiro et al., 1997a, 1997b that used the same apparatus) represents a sans-serif font. In our experiment, we used Lucida Sans, which upon inspection looked relatively similar to the font represented in Fig. 1 of the original experiment. We are not aware of any study investigating whether the font type modulates the effect. In addition, it is unclear in which direction the font may modulate the effect. The majority of current works on attentional blink report the font used for the stimulus. A direct comparison of the font depicted in the original study and the font used in our experiment is available in the Supplemental Material, Part 4 (Figure S4).
- ***Differences in the pause after the fixation point.*** Inspection of Fig. 1 of the original paper indicates that the stimulus was preceded by a “fixation spot,” but the paper does not report the duration of the interval separating this fixation spot and the first letter of the stimulus’s stream. We set this duration to 283.33 ms (refresh rate 60 Hz) or 242.85 ms (refresh rate 70 Hz). This seems to be a theoretically irrelevant difference: to the best of our knowledge, there are no studies or reviews mentioning the duration of this pause in the definition of the attentional blink (e.g., see MacLean & Arnell, 2012).
- ***Differences in luminance of the laboratory room.*** The authors of the original paper did not report the luminance of the room. We opted to run the experiment with dim light. In addition, the dim light was not directly hitting the monitor used in the experiment.
- ***Differences in the number of letters following the probe***^4^***.*** In the original experiment, “Eight letters always succeeded the target” (Raymond et al., 1992, p. 852), referring to the number of post-target items. This detail was mentioned in Experiment 1 but not in Experiment 2. Because of this choice, when the probe was presented at lag 8, the probe was also the last letter of the stream. If the probe is not followed by a backward masking trail, the detection of the probe reaches ceiling (Giesbrecht & Di Lollo, 1998, Fig. 2B). Here, a minimum of two letters always followed the probe. Notably, the masking effect of the trail following the probe is independent of the number of maskers in the trail (see Giesbrecht & Di Lollo, 1998, Experiment 1, Figure 2A).

#### Code for running the experiment

The program for running the experiment was written in MATLAB with the Psychophysics Toolbox extensions (Kleiner et al., 2007). It can be accessed via the preregistration document (https://osf.io/hp9nk/).

#### The dependent variable

The attentional blink paradigm uses a “yes/no” task (probe detection) and a forced choice task (target identification). In the original study, the authors performed statistics on both and labeled the former “correct probe detection.” The “correct probe detection” was conditional on the correct identification of the target (i.e., trials with incorrect target identification were excluded from the statistical analysis). In addition, “correct probe detection” included hits only, i.e., the proportion of “yes, probe present” responses when the probe was actually presented (Raymond, personal communication). In the statistical analysis reported, we calculated “correct probe detection” as in the original paper. Here, we did not analyze the “target identification errors” like in the original paper. However, the Supplemental Part 7 includes one table and one figure that compare errors observed in Raymond et al. (1992) with those observed here.

- ***Statistical modeling.*** Raymond et al. (1992; Raymond, personal communication) screened out participants who were guessing (i.e., with an overall false alarm rate in probe detection larger than 45%). This criterion was not explicitly stated in the original paper. However, we chose to apply it and thereby excluded two participants. Our final sample size was *N* = 98.

A first analysis was conducted using a repeated-measures ANOVA as in Raymond et al. (1992). The main effects of condition (control vs. experimental, as a within-participant factor), lag (0 to 8, as another within-participant factor), and their interaction on correct probe detection were thus examined.

A more detailed analysis was conducted using generalized mixed-effects linear models to obtain a quantitative description of the phenomenon. As the response variable consisted of a series of dichotomous responses (“yes/no” answers to probe detection), mixed-effects logistic regressions were used (Baayen, 2008; Jaeger, 2008), which allowed us to model both fixed and random effects. Participants were entered as random effects (with random intercepts). Condition and lag were entered as fixed effects. Refresh rate was controlled for by entering it as another fixed effect. Lag 0 was excluded because in the experimental condition its accuracy is bound to have no variability.

Logistic mixed-effects linear models were fitted using the “brms” package (Bürkner, 2017) of R software (R Core Team, 2017), which implements Markov chain Monte Carlo (MCMC) Bayesian estimation methods using the Stan programming language. All models were fitted using four chains of 5000 iterations each. The Rhat index was below 1.01 for all model parameters reported below, indicating perfect convergence.

The evidence of the effects was assessed with the widely applicable information criterion (WAIC; Watanabe, 2010) and the evidence ratio based on it (Burnham, Anderson, & Huyvaert, 2011). The latter indicates the relative likelihood of a model being better than a competitor given the data (when the models differ by one effect, it indicates the strength of evidence in favor of or against that effect).

#### Definition and use of prior knowledge

Prior knowledge was taken from the findings reported in Figure 3A of Raymond et al. (1992). Normal distributions were used. The observations of the “correct probe detection” were treated as estimated probabilities. As we used logistic regressions, model coefficients were formalized on the logit scale. Unfortunately, the figure did not present any information about the uncertainty of the estimates (e.g., standard errors). This could be approximated from the parameters of the binomial distribution, but this would neglect correlations among repeated responses. Therefore, to estimate plausible uncertainty, we used our participants from Experiment 1, who were randomly resampled with *N* = 10 for 10,000 iterations. At each iteration, model coefficients were estimated with maximum likelihood. The SDs of these coefficients’ distributions were used for the prior distributions. Uninformed default priors were used for all other model parameters. Parameters can be found in Table 1.

**Table 1:** Parameters of informed prior distributions for model coefficients (on the logit scale) derived from Raymond et al. (1992). Normal distributions were used. Baseline levels are “control” for condition, and “lag 1” for lag

Coefficient	Mean	Estimated SD
Intercept	2.20	.44
Lag 2	.12	.38
Lag 3	−.46	.37
Lag 4	.25	.41
Lag 5	.00	.44
Lag 6	.00	.44
Lag 7	.00	.47
Lag 8	1.69	.40
Experimental	−.68	.47
Experimental × Lag: Lag2	−1.23	.45
Experimental × Lag: Lag3	−1.18	.50
Experimental × Lag: Lag4	−1.56	.51
Experimental × Lag: Lag5	−1.03	.57
Experimental × Lag: Lag6	−.42	.62
Experimental × Lag: Lag7	.68	.66
Experimental × Lag: Lag8	.68	.59

*Note*. Coefficients are on the logit scale because they were formalized to be used in logistic regression models. Intercept corresponds to the performance at lag 1 in the control condition

To assess whether the use of informed priors leveraged the posterior estimates, the models were also fitted using uninformed default priors. The posterior distributions obtained using informed versus uninformed priors were virtually identical (see the “sensitivity analysis” in Supplemental Material, Part 5). To consider only evidence brought by the data, however, the evidence ratios reported in the Results section of the current experiment refer to models using uninformed priors. Nonetheless, the posterior distributions that we subsequently examined came from models including the informed priors.

### Results

#### Descriptive statistics and ANOVA

Raw data and the analysis scripts are available here: https://figshare.com/s/ef5a62fd5fa70c214b62. Table 2 reports the descriptive statistics (mean and standard deviations) for correct probe detection averaged by participant, separately by condition and lag. The mean false alarm rate was 9% for the control condition (range 0% to 38%) and ~13% for the experimental condition (range 0% to 47%). In the experimental condition, target identification was 85.8% in probe-absent trials and 84.6% in probe-present trials. In Raymond et al. (1992) these values were, respectively, 78.0% and 75.3%^5^. Additional results about the target identification are reported in the Supplemental Part 7.

**Table 2:** Means (and SD) of the proportions of correct probe detection in Experiments 1 (*N* = 98) and 2 (*N* = 29)

Lag	*Experiment 1*		*Experiment 2*	
	Condition		Condition	
	Control	Experimental	Control	Experimental
Lag 0	.86 (.19)	.99 (.02)	.92 (.10)	.99 (.04)
Lag 1	.79 (.21)	.51 (.31)	.85 (.14)	.46 (.27)
Lag 2	.75 (.20)	.33 (.26)	.77 (.17)	.28 (.26)
Lag 3	.74 (.21)	.33 (.26)	.78 (.20)	.30 (.29)
Lag 4	.74 (.20)	.43 (.28)	.74 (.17)	.42 (.26)
Lag 5	.75 (.22)	.57 (.26)	.75 (.18)	.54 (.26)
Lag 6	.75 (.22)	.66 (.27)	.78 (.13)	.62 (.25)
Lag 7	.73 (.24)	.70 (.26)	.80 (.15)	.70 (.24)
Lag 8	.71 (.23)	.70 (.27)	.76 (.20)	.65 (.23)

To replicate the analysis by Raymond et al. (1992), we first conducted a two-way 2 (condition: control vs. experimental) × 9 (lag: 0 to 8) repeated-measures ANOVA, with the mean proportions of correct probe detection as the dependent variable. Both main effects and the interaction were significant: condition, *F*(1,1745) = 249.34, *p* < .001; lag, *F*(8,1745) = 49.29, *p* < .001; interaction, *F*(8,1745) = 33.28, *p* < .001. The main effect of refresh rate did not reach statistical significance, *F*(1,1744) = .92, *p* = .34. Furthermore, it did not moderate the two-way interaction between condition and lag, *F*(8,1727) = .53, *p* = .84.

#### Bayesian logistic mixed-effects models

Unsurprisingly, the Bayesian analysis suggested extremely strong evidence in favor of both main effects and the interaction: for condition, evidence ratio > 1.0e+10 (ΔWAIC = 867.30); for lag, evidence ratio > 1.0e+10 (ΔWAIC = 305.20); for the interaction, evidence ratio > 1.0e+10 (ΔWAIC = 380.80). Figure 1 shows the posterior estimated accuracies in the probe identification as a function of condition and lag.

Subsequently, we entered refresh rate (60 vs. 70 Hz) as an additional factor in the model. Very weak evidence emerged in favor of its main effect: evidence ratio = 2.12 (ΔWAIC = 1.50); the estimated coefficient B = −.19, SE = .21, suggested a negligible drop in correct probe detection at 70 Hz in comparison to 60 Hz frequency. There was evidence against a three-way interaction of refresh rate with condition and lag, evidence ratio = .09 (ΔWAIC = −4.90). Despite the evidence against the three-way interaction, this result must be taken with caution, as the study was not designed to have enough power for the difference in refresh rate, which was intentionally kept to a minimum. Descriptive statistics for the two levels of the refresh rate are reported in the Supplemental Material, Part 2.

#### Replication assessment

We assessed whether our results replicated those of the original experiment in two ways: statistically and subjectively (i.e., asking attentional blink experts their opinion).

##### Statistical assessment

All estimated probabilities (see Fig. 1) excluded the corresponding prior mean values from their 95% Bayesian credible intervals (BCI, calculated with the percentile method), except in the experimental condition at lags 5 and 6. This may simply reflect a difference in the overall accuracy (i.e., the intercept of the model), however, without affecting the magnitude or shape of the blink. Nonetheless, Supplemental Material, Part 1, reports all model coefficients, showing that most of them (not only the intercept) deviated substantially from the priors.

To examine the magnitude and shape of the blink, we compared the differences in accuracy between control and experimental condition at each lag. As the response variable was binomial, odds ratios (OR) were used (for those unfamiliar with OR, Cohen’s *d* can be derived as *d* = Log(OR)*(√(3)/π); Hasselblad & Hedges, 1995). Odds ratios are reported in Table 3, along with their comparisons against the priors by Raymond et al. (1992). Overall, the magnitude of the differences seems comparable. However, at lags 1, 2, and 3, the differences were larger in our Experiment 1 than in the original study, and the opposite was true for lags 4, 5, and 6. At lags 7 and 8, the blink can be considered to have concluded in both experiments. The evidence ratios (Table 3) use the posterior distributions to formally test the hypotheses that our effect sizes differed from those of Raymond et al. (1992). These evidence ratios were computed as relative probabilities of the estimated effects lying above rather than below (or vice versa) the corresponding points in Raymond et al. (1992). To summarize, it seems that the magnitude and width of the blink were very similar to those of Raymond et al. (1992), but systematically early by about one lag.

**Table 3:** Odds ratio of the control vs. experimental comparison at each lag, and evidence ratio by which they differ from the prior values (from Raymond et al., 1992) as indicated in the “target” hypothesis

	Odds ratio Experiment 1	Odds ratio Experiment 2	Prior odds ratio (Raymond et al., 1992)	Target hypothesis	Evidence ratio in Exp 1	Evidence ratio in Exp 2
Lag 1	3.41	5.02	1.97	> prior	>10,000.00	>10,000.00
Lag 2	8.77	10.41	6.75	> prior	122.46	57.82
Lag 3	8.05	10.37	6.42	> prior	57.48	148.25
Lag 4	5.12	4.76	9.49	< prior	>10,000.00	9999.00
Lag 5	2.63	2.84	5.53	< prior	>10,000.00	4999.00
Lag 6	1.55	2.31	3.00	< prior	>10,000.00	9.05
Lag 7	1.21	1.72	1.00	-	-	-
Lag 8	1.16	1.72	1.00	-	-	-

*Note*: Odds ratio = 1.00 indicates null effect (i.e., null difference between control and experimental condition). Evidence ratio = 1.00 indicates that the odds ratio estimated in the present experiment(s) was equal to the corresponding one in Raymond et al. (1992). For lags 7 and 8, evidence target hypotheses were not tested, as the attentional blink seemed to have ended there

The lag-1 sparing we observed seemed smaller than that of Raymond et al. (1992), supporting the idea of a general difference in timing. We measured lag-1 sparing as the difference in accuracy between lag 1 and the minimum of the blink (which in Raymond et al., 1992, occurs at lag 3) in the experimental condition, minus the baseline (control condition). Based on prior knowledge, the lag-1 sparing estimated from Raymond et al. (1992) was odds ratio = 3.25; translated into accuracy, Δ = 30%. In our Experiment 1, it was odds ratio = 2.36, 95% BCI (1.80, 3.10); translated into accuracy, Δ = 18%, 95% BCI (10%, 27%). In brief: a smaller sparing.

An additional analysis was conducted using the parameterization suggested by Cousineau et al. (2006). Unsurprisingly, the estimated “amplitude” and “width” of the attentional blink in our Experiment 1 were very similar to those from Raymond et al. (1992). Conversely, “lag-1 sparing” and “minimum” were smaller. All details are reported in the Supplemental Material, Part 3.

From a statistical point of view, the results of our experiment largely replicated the specific pattern of results of Raymond et al. (1992), but they also showed some differences. Specifically, the differences concerned the absolute level of performance and a difference in the onset, peak, and offset of the blink that were found one lag earlier in comparison to those observed by Raymond et al. (1992). The differences in timing may be related to the overall lower performance in our study. This is examined in the Supplemental Material, Part 6 (participants above vs. below the median accuracy in Experiment 1, however, had similar timings).

##### Subjective assessment

Subsequently, we contacted a group of 33 experts on the attentional blink^6^. The group included the authors of the original paper as well as the authors of highly cited empirical papers (i.e., more than 100 citations in Google Scholar) investigating the attentional blink. The first and last authors of these papers were contacted. Experts were presented with a graph representing the results of our experiment together with the original results (nearly identical to the one reported in Fig. 1) and were asked to rate the similarity through a Google form and by means of five Likert scale questions ranging from 1 (i.e., “Not at all replicated”) to 7 (i.e., “Fully replicated”). Eleven experts returned responses. Questions and descriptive statistics of the responses are reported in Table 4.

**Table 4:** Replication rating by experts of attentional blink. Experts responded on a Likert scale of 1 to 7 (1, “Not at all replicated”; 7, “Fully replicated”)

Question	Mean	Median	SD	Min	Max
“Were the results of the control condition replicated?”	6	6	1	4	7
“Were the results of the experimental condition replicated?”	6.1	6	0.8	5	7
“Was the relationship between control and experimental condition replicated?”	6.8	7	0.4	6	7
“Was the lag-1 sparing replicated?”	6.1	7	1.4	3	7
“Were the results replicated (overall rating)?”	6.4	6	0.7	5	7

Overall, experts stated that our data replicated almost identically the results of the original experiment, both for the control condition, the experimental condition, the relationship between control and experimental conditions, and the lag-1 sparing.

## Experiment 2

Since some results provided in the original report were not entirely replicated in Experiment 1, we decided to perform Experiment 2 as a direct replication of Experiment 1. This aimed to clarify whether the results of our first experiment were consistent and accurate. Experiment 2 was conducted by an independent research group based in a different country (United Kingdom). The same procedure and materials as our first experiment were used but the experiment was run on a new set of participants with a different experimenter, in a different laboratory. Results of Experiment 2 were then compared to the results of Experiment 1 and the original results of Raymond et al. (1992).

### Methods

#### Participants

Thirty undergraduate students (mean age = 26.20, SD = 3.10, range = 21 to 33 years, females = 21) from Liverpool John Moores University, UK, participated voluntarily, without receiving payment or course credit. They were tested by a research group of the same university. As described in the “replication of a replication” preregistration form, the sample size was determined to establish whether the results collected in Liverpool would closely resemble those reported in our Experiment 1 (rather than those of Raymond et al., 1992). A bootstrapping procedure based on randomly resampling participants from Experiment 1 suggested that a sample size of about 30 would lead to 95% confidence intervals clearly excluding all original estimates by Raymond et al. (1992) (except lags 5 and 6 in the experimental condition, which could not be excluded even with *N* = 98). A sample size of 30 was also enough to clearly exclude a near ceiling effect (i.e., accuracy higher than .85 as in Raymond et al., 1992) in the control condition. Because of an error by the experimenter, one male participant ran only the control condition, and he had to be excluded from the analysis. The final sample included 29 participants.

#### Apparatus, materials, and procedure

The study was approved by the local ethics committee. The software, materials, and procedure used in Experiment 2 were identical to those in Experiment 1, whereas the hardware apparatus was different. Stimuli were generated by a MacBook Pro computer, connected to a Philips I70S4 17-inch color LCD monitor. Unlike Experiment 1, the monitor refresh rate was 60 Hz for all participants. Also, the Liverpool research group calibrated the stimuli according to the size and luminance reported in the original experiment by Raymond et al. (1992).

#### Statistical analysis and definition of prior knowledge

The analysis was conducted as in Experiment 1, with the same prior distributions (Table 1), but with slightly larger SDs (i.e., SD = 1.00 for all coefficients). Larger prior distributions were used for two reasons. First, the findings from Experiment 1 failed to confirm the prior estimates from Raymond et al. (1992). Second, the sample size in Experiment 2 was smaller, meaning that a strong set of priors could have had unwarranted leverage on the posterior estimates.

### Results

Raw data and the analysis scripts are available here: https://figshare.com/s/ef5a62fd5fa70c214b62. Descriptive statistics of the correct probe detection (with standard deviations expressing inter-individual variability) calculated separately by condition and lag are reported in Table 2. In the experimental condition, target identification was 77.7% in probe-absent trials and 78.4% in probe-present trials (see Supplemental Part 7 for further results). Unsurprisingly, the two-way 2 (condition: control vs. experimental) × 9 (lag: 0 to 8) repeated-measures ANOVA on proportions of probe detection as the dependent variable revealed that both main effects and the interaction were significant: main effect of condition, *F*(1,512) = 181.55, *p* < .001; main effect serial position of probe, *F*(8,512) = 23.80, *p* < .001; interaction, *F*(8,512) = 12.56, *p* < .001. The mean false alarm rate was ~10% for the control condition (range 1% to 30%) and ~10% for the experimental condition (range 0% to 53%). No participant showed an overall false alarm rate greater than 45%.

The Bayesian analysis with evidence ratios brought extremely strong evidence in favor of all three effects: main effect of condition, evidence ratio > 1.0e+10 (ΔWAIC = 377.7); main effect of lag, evidence ratio > 1.0e+10 (ΔWAIC = 96.1); interaction between condition and lag, evidence ratio > 1.0e+10 (ΔWAIC = 84.5). Details on the posterior distributions of all model coefficients can be found in Supplemental Material, Part 1.

### Replication assessment

Using the same procedure as Experiment 1, we reported the estimated posterior probabilities of correct probe detection as a function of condition and lag (see Fig. 1). Once again, most posterior distributions of the estimated accuracies exclude the prior mean values from their 95% credible intervals. Because the estimates from Experiment 2 were nearly identical to those observed in Experiment 1, we did not seek further subjective evaluations from attentional blink experts.

With regard to the effect sizes comparing probe detection accuracy in the experimental versus control condition at each lag, results are reported in Table 3. The target hypotheses were kept identical to Experiment 1. Evidence ratios confirmed that all differences were substantial, except at lag 6. Once again, the onset, peak, and offset of the phenomenon were early by at least one lag when compared to the original study (Raymond et al., 1992). Regarding the lag-1 sparing, we estimated it from Raymond et al. (1992) as odds ratio = 3.25 (translated into accuracy, Δ = 30%). In our Experiment 2, odds ratio = 2.07, 95% BCI (1.22, 3.45); translated into accuracy, Δ = 14%, 95% BCI (-3%, 29%).

## General discussion

Here, we replicated a classic study of cognitive psychology—the seminal Experiment 2 of Raymond et al. (1992) on attentional blink—while adopting several of the currently advocated and endorsed research practices (e.g., large power, new statistical standards, accurate estimation of parameters and their confidence intervals, preregistration, sharing of data and materials). Experiment 1 replicated the original experiment, performed with a much larger sample size. Experiment 2 is a direct replication of Experiment 1 and was performed to assess to what extent the adoption of the abovementioned research practices enabled us to replicate this experiment’s findings. Rather than testing the “significance” of the blink, here we aimed to provide an accurate picture of the phenomenon under the conditions of our experiments, and to establish whether it would be the same across our Experiments 1 and 2.

The results of Experiment 1 revealed the blink (i.e., the drop/recovery in performance in the experimental condition in comparison to the control condition). However, the picture of the blink returned by the experiment was slightly different from that reported in the original one. Specifically, the performance level was systematically worse than in the original experiment (about 15% worse in the control condition, and 2% to 35% in the experimental condition). Furthermore, we observed differences in some of the fundamental characteristics that describe the attentional blink. Although the magnitude of the blink (i.e., the difference between experimental and control conditions) was generally comparable to that reported in the original study (see Table 3), its timing seemed different. Specifically, the onset, peak, and offset of the blink were about one lag earlier in comparison to the original experiment. It is possible that the earlier blink emerged because here the performance of the participants on the probe was worse than that observed in the original experiment (see Supplemental Material, Part 6). In any case, one consequence of this earlier blink was that the lag-1 sparing (i.e., a higher than expected performance when the probe immediately followed the target) was smaller here than in the original experiment. Another difference was the lack of a convergence at ceiling of the performance in the control and experimental conditions at lags 7 and 8, as the observed convergence was well below the performance at the starting point, lag 0. As far as lag 8 is concerned, this is likely due to the fact that Raymond et al. (1992) did not mask the probe at lag 8 and thus gathered a ceiling performance for the longest lag in both the experimental and the control conditions (see Giesbrecht & Di Lollo, 1998). In other ways, however, the results of the original and the present experiments were also very similar. This is the case of the maximum size of the effect: in the original experiment the magnitude of the peak of the blink had an effect size (odds ratio) of 9.49; the effect sizes we observed here were 8.77 (Experiment 1) and 10.41 (Experiment 2). Notably, in recent direct replications, the effect sizes of the replications are usually smaller or much smaller than the original effect sizes (e.g., Open Science Collaboration 2015; Camerer et al., 2018). This was not our case.

All of the abovementioned differences were statistically relevant, as suggested by the fact that most data points of the original experiments fell outside the 95% credibility intervals, and the evidence ratios of differences were generally large. Despite this finding, experts on attentional blink judged these differences to be negligible and stated that our results replicated the original results in all respects. This highlights the discrepancy between a statistical criterion and an expert-based, qualitative and theoretically motivated criterion. Statistical analysis suggested that the results observed in Experiment 1 differed in a series of aspects from the original results, yet experts did not take these differences into account.

Experiment 2 was conceived as a direct replication of our Experiment 1 but performed by another research group. This research group was given a specific number of participants that needed to be targeted, a number that we estimated should be large enough to replicate the results of Experiment 1 if these were representative of the true effect. Experiment 2 replicated nearly identically the results of Experiment 1, highlighting that a step-by-step replication of the method and procedure of an attentional blink experiment can lead to a *point-by-point replication* of the results, a noteworthy finding to advance the science of psychology. The overlap of the results gathered in Experiments 1 and 2 is quite striking given that the two experiments recruited different groups of participants from different countries who spoke different languages and were run by different experimenters in different labs. This overlapping supports the recent observation that, for truly existing effects, testing different samples is not a sufficient reason to explain a difference in the outcome of two studies (Klein et al., 2018). Here, the results seem even more remarkable given that the design of the experiment is a multifactorial one and articulated on several levels. In an additional analysis, we also assessed whether the differences observed between our results and the original ones could merely reflect the inaccuracy of the original report due to their limited sample size (i.e., *N* = 10, Raymond et al., 1992). This comparison is reported in the Supplemental Material, Part 3, and for the sake of simplicity it was based on the parameterization of the attentional blink suggested by Cousineau et al. (2006). This analysis revealed once again that the blink amplitude (magnitude) and width observed by Raymond et al. (1992) were fully compatible with those we could observe in our experiments (if these had been run on 10 participants). However, the same was not true for the lag-1 sparing and minimum of the blink, which in Raymond et al. (1992) seemed rather exceptional if our results were assumed to be the true ones.

In our replication project, we sought to closely follow the description of the experiment reported in the original study. Undoubtedly, the practice of sharing (e.g., sharing the experiment code) would solve much of the burden of replicating an original experiment. In addition, if the instructions given to the participants were also included (e.g., Zwaan et al., 2018), the issue of replicating an original experiment would be limited to the calibration of the stimulus for the apparatus that the replicator was using. Sharing the code of an experiment would only improve the replicability of experiments in experimental psychology. Similarly, sharing data and the analysis scripts would also increase the replicability of an experiment, revealing all the successive operations performed on the data. We believe that, altogether, the sharing of the materials of an experiment is a no-cost, benefits-only action that could improve the reproducibility of experimental psychology. The verbal descriptions reported in the experiments are, in most cases, missing some details even when they are written by the most scrupulous scientist.

Because the present study is a replication of an original experiment, it is legitimate to ask whether the original findings were replicated. Evaluating whether the results were replicated is a complex issue that requires not only data but also an expert’s eye. Here, original results did not fall into the credibility intervals calculated in Experiment 1 and Experiment 2. However, experts judged this fact to be negligible and evaluated our results as having replicated the original findings very well. In fact, even differences that are strongly compelling from a statistical point of view (e.g., with evidence ratio > 1000) may still be trivial from a theoretical standpoint if they do not modify the overall account of a phenomenon of interest. It is likely that experts evaluated the relation between control and experimental performance in the original and in our Experiment 1 rather than the absolute level of performance. Nonetheless, Experiment 2 replicated nearly identically the results observed in Experiment 1, demonstrating empirically that it is possible to replicate the results of the experiment point by point, independently of the participants recruited for the experiment (Klein et al., 2018). We also think it demonstrates empirically that, in well-controlled studies, the comparison of experimental results can be made globally (across laboratories) and not only locally (i.e., within a single study and laboratory), as we currently do in contemporary psychological research.

Together with the magnitude of the lag-1 sparing, a major difference between our results and the original ones is the overall accuracy of the participants. When the original experiment appeared, the discussion about the phenomenon concentrated on whether the phenomenon was a sensory or an attentional one. Broadbent and Broadbent (1987) and Weichselgartner and Sperling (1987) were the first to observe the blink but used rather complex stimuli (the former) or task (the latter) that could not disentangle the issue. Raymond et al.’s (1992) experiment had the advantages of simplifying stimulus and task and including a control condition. When the paper appeared, the high detection performance in the control condition (i.e., greater than 90%) was regarded by the original authors as a signal that the blink was an attentional and not a sensory phenomenon. This was also supported by successive data with participants scoring, on average, 90% hits in all the experiments that included a control-like condition (i.e., see Raymond et al., 1992, Experiment 3; Shapiro et al. 1994, Experiments 1, 2, 3A, 3B, 4, 5A, 5B; Raymond et al., 1995, Experiment 1). Notably, Raymond et al. (1992) stressed that this high performance was obtained with “less-practiced subjects” (Raymond et al., 1992, p. 853) than the “high-practiced subjects” of preceding studies (e.g., Weichselgartner & Sperling, 1987). Indeed, the target does not forward-mask the probe, and in the control condition there is no drop in performance after the target. However, in our replication the probe detection was not that high all the way through, revealing a lower signal strength than in the original experiment. Here, the average performance in the control condition was ~75%, showing that the task was, from a sensory point of view, much more demanding than that depicted by the original study. Such a drop in performance may suggest that some of the characteristics of the original experiment that we were unable to replicate may perhaps modulate both the signal strength and the temporal characteristics of the blink, and that they should be taken into account by current computational models depicting this attentional phenomenon (e.g., Wyble, Bowman, & Nieuwenstein, 2009; Taatgen, Juvina, Schipper, Borst, & Martens, 2009; Olivers & Meeter, 2008).

Whether a result has been replicated depends upon the type of phenomena evaluated. The variety of phenomena we investigate in experimental psychology is rather wide. Some effects are large and robust (e.g., the susceptibility to classic illusions of visual perception) whereas others are small and less robust (e.g., a behavior in response to a given social context that might change from country to country or from year to year). The attentional blink falls within the class of robust effects. If we take as representative of the blink the difference in performance at lag 2 between the control and experimental conditions, such a difference can be observed with as few as ten participants (as in the original experiment, Raymond et al., 1992). However, the nearly complete overlap between the results of Experiment 2 with Experiment 1 suggests that the replicability of robust effects will benefit too from higher research standards. Here we replicated not only the blink, but also the quantitative characteristics of the blink, across two experiments. Higher standards mean, among the various possibilities, large statistical power^7^ and an accurate description of phenomena that emphasizes the estimation of effects with uncertainty (Kruschke & Liddell, 2015), rather than the simplified reject/not-reject dichotomous choice of the traditional NHST approach (McElreath, 2016).

Although the blink was evident in the original and in both of our experiments, we observed some differences between the results of the present studies and the original ones. If we assume that the original results can be directly compared with those of our two experiments in terms of the characteristics of the phenomenon, we may conclude that, more than 25 years later, the blink has changed shape. Today’s participants were less accurate, and today’s blink was one lag earlier than the blink of 25 years ago. Why this happened, however, can only be speculated. It is possible that the sample size in the original experiment was insufficient to quantify all the aspects of the blink with precision. Our data suggest that a minimum of 30 participants is necessary to provide a veridical picture of the blink. For this reason, the picture of the effect returned by the original paper may not have been entirely veridical (see also Etz & Vandekerckhove, 2016; Morey & Lakens, 2016). By the same token, the present results might give a more precise estimation of the blink observed in Raymond et al. (1992). We considered this possibility and, by sampling our data, we simulated running the experiment with ten participants only (see Supplemental Material, Part 3). This analysis explains some of the differences but not all of them. It is also possible that the lack of overlap between the original results and the present results may be due to minor differences in the methods and theoretically relevant details that were “lost in translation”—details that, somehow, accounted for the differences we observed between our results and the original one. Unfortunately, there is no way to test this hypothesis.

In sum, in the present study, we implemented a set of currently advocated research practices (i.e., preregistration, large statistical power, availability of research data and materials, new statistical standards) for the direct replication of a classic paradigm in experimental psychology, the attentional blink (Raymond et al., 1992). The present study showed empirically that the adoption of the aforementioned research practices enables us to raise the current standards of psychological research by providing an account of a psychological phenomenon that could be replicated across two experiments, regardless of whether the data were collected in different countries, by different laboratories and experimenters, and with different participants. This assertion was empirically supported by the almost perfectly overlapping results of Experiments 1 and 2.

## Electronic supplementary material

ESM 1 (DOCX 3752 kb)

## Footnotes

1 https://featuredcontent.psychonomic.org/alpha-beta-blink-synchronicity-between-brain-oscillations-and-the-attentional-blink/

2 Some Apple Macintosh II computers (i.e., the computer used by Raymond et al., 1992) were equipped with a 66 Hz monitor.

3 The original study reports the following: “Responses were recorded verbally and were recorded by an experimenter with the aid of a second computer. The experimenter was unaware of the correct responses of all trials.” We interpreted this by sitting the experimenter in a position where she could not see the monitor and the stimulus.

4 We thank Raymond Klein for pointing out this difference between our experiment and the original one.

5 Values were calculated from Figure 3B of Raymond et al. (1992).

6 For privacy reasons we prefer these scientists to remain anonymous.

7 It is noteworthy that a large power can be obtained with relatively little cost in many experiments in psychology.

## References

[CR1] American Psychological Association. (2010). Publication manual of the American Psychological Association (5th ed.). Washington, DC: American Psychological Association.

[CR2] Baayen RH (2008). *Analyzing linguistic data: A practical introduction to statistics using R*.

[CR3] Bakker, M., & Wicherts, J. M. (2011). The (mis)reporting of statistical results in psychology journals. *Behavior Research Methods, 43*(3), 666–678. 10.3758/s13428-011-0089-5 10.3758/s13428-011-0089-5 PMC3174372 21494917

[CR4] Bakker, M., & Wicherts, J. M. (2014). Outlier removal, sum scores, and the inflation of the type I error rate in independent samples t tests: The power of alternatives and recommendations. *Psychological Methods, 19*(3), 409–427. 10.1037/met0000014 10.1037/met0000014 24773354

[CR5] Broadbent DE, Broadbent MH (1987). From detection to identification: Response to multiple targets in rapid serial visual presentation. Perception & Psychophysics.

[CR6] Bürkner, P. C. (2017). Brms: an R package for Bayesian multilevel models using Stan. *Journal of Statistical Software, 80*, 1-28. 10.18637/jss.v080.i01

[CR7] Burnham, K. P., Anderson, D. R., & Huyvaert, K. P. (2011). AIC model selection and multimodel inference in behavioral ecology: Some background, observations, and comparisons. *Behavioral Ecology and Sociobiology, 65*, 23-35. 10.1007/s00265-010-1029-6

[CR8] Camerer CF, Dreber A, Holzmeister F, Ho TH, Huber J, Johannesson M (2018). Evaluating the replicability of social science experiments in Nature and Science between 2010 and 2015. Nature Human Behaviour.

[CR9] Chambers C (2017). *The seven deadly sins of psychology: A manifesto for reforming the culture of scientific practice*.

[CR10] R Core Team (2017). *R: A language and environment for statistical computing*. R Foundation for Statistical Computing, Vienna, Austria. Retrieved from: https://www.R-project.org/

[CR11] Cousineau, D., Charbonneau, D., & Jolicoeur, P. (2006). Parameterizing the attentional blink effect. *Canadian Journal of Experimental Psychology, 60*(3), 175-189. 10.1037/cjep2006017 10.1037/cjep2006017 17076433

[CR12] Cowan, N. (2018). Experimental Psychology Generally, and the Journal Today. *Journal of Experimental Psychology: General, 147*(4), 459-461. 10.1037/xge0000439 10.1037/xge0000439 29698024

[CR13] Cumming, G., Fidler F., Leonard, M., Kalinowski, P., Christiansen, A., Kleinig, A., Lo N., & McMenam (2007). Statistical reform in psychology: Is anything changing? *Psychological Science, 18*, 230-232. 10.1111/j.1467-9280.2007.01881.x 10.1111/j.1467-9280.2007.01881.x 17444919

[CR14] Dux, P. E., & Marois, R. (2009). The attentional blink: A review of data and theory. *Attention, Perception, & Psychophysics, 71*(8), 1683-1700. 10.3758/APP.71.8.1683 10.3758/APP.71.8.1683 PMC2915904 19933555

[CR15] Etz A, Vandekerckhove J (2016). A Bayesian perspective on the reproducibility project: Psychology. PLOS ONE.

[CR16] Giesbrecht B, Di Lollo V (1998). Beyond the attentional blink: visual masking by object substitution. Journal of Experimental Psychology: Human Perception and Performance.

[CR17] Giofrè, D., Cumming, G., Fresc, L., Boedker, I., & Tressoldi, P. (2017). The influence of journal submission guidelines on authors’ reporting of statistics and use of open research practices. *PLOS ONE, 12*(4), e0175583. 10.1371/journal.pone.0175583 10.1371/journal.pone.0175583 PMC5393581 28414751

[CR18] Hasselblad V, Hedges LV (1995). Meta-analysis of screening and diagnostic tests. Psychological Bulletin.

[CR19] Hulme C, Maughan S, Brown GD (1991). Memory for familiar and unfamiliar words: Evidence for a long-term memory contribution to short-term memory span. Journal of Memory and Language.

[CR20] Jaeger, T. F. (2008). Categorical data analysis: Away from ANOVAs (transformation or not) and towards logit mixed models. *Journal of Memory and Language, 59*, 434-446. 10.1016/j.jml.2007.11.007 10.1016/j.jml.2007.11.007 PMC2613284 19884961

[CR21] Klein RA, Vianello M, Hasselman F, Adams BG, Adams RB, Alper S (2018). Many Labs 2: Investigating variation in replicability across samples and settings. Advances in Methods and Practices in Psychological Science.

[CR22] Kleiner, M., Brainard, D., Pelli, D., Ingling, A., Murray, R., & Broussard, C. (2007). What’s new in Psychtoolbox-3? *Perception*, *36*(Suppl. 1), 14. 10.1177/03010066070360S101

[CR23] Kruschke JK (2014). *Doing Bayesian data analysis: A tutorial with R, JAGS, and Stan*.

[CR24] Kruschke, J. K., & Liddell, T. M. (2015). The Bayesian new statistics: Two historical trends converge. *SSRN Electronic Journal*, 2606016.

[CR25] Lindsay DS, Simons DJ, Lilienfeld SO (2016). Research preregistration 101. Observer.

[CR26] MacLean MH, Arnell KM (2012). A conceptual and methodological framework for measuring and modulating the attentional blink. Attention, Perception, & Psychophysics.

[CR27] Martens, S., & Wyble, B. (2010). The attentional blink: Past, present, and future of a blind spot in perceptual awareness. Neuroscience & Biobehavioral Reviews, 34(6), 947-957. 10.1016/j.neubiorev.2009.12.005 10.1016/j.neubiorev.2009.12.005 PMC2848898 20025902

[CR28] McElreath R (2016). *Statistical rethinking: A Bayesian course with examples in R and Stan*.

[CR29] McLaughlin, E., Shore, D., & Klein, R. (2001). The attentional blink is immune to masking-induced data limits. *The Quarterly Journal of Experimental Psychology, 54*, 169-196. 10.1080/02724980042000075 10.1080/02724980042000075 11216315

[CR30] Morey, R. D., & Lakens, D. (2016). Why most of psychology is statistically unfalsifiable. Manuscript submitted for publication. Retrieved from https://github.com/richarddmorey/psychology_resolution/blob/master/paper/response.pdf.

[CR31] Olivers CN, Meeter M (2008). A boost and bounce theory of temporal attention. Psychological Review.

[CR32] Open Science Collaboration. (2015). Estimating the reproducibility of psychological science. *Science*, *349*(6251), aac4716. 10.1126/science.aac4716 10.1126/science.aac4716 26315443

[CR33] Pashler, H., & Harris, C. R. (2012). Is the replicability crisis overblown? Three arguments Examined. *Perspectives on Psychological Science, 7*, 531-536. 10.1177/1745691612463401 10.1177/1745691612463401 26168109

[CR34] Popple AV, Levi DM (2007). Attentional blinks as errors in temporal binding. Vision Research.

[CR35] Raymond, J. E., Shapiro, K. L., & Arnell, K. M. (1992). Temporary suppression of visual processing in an RSVP task: An attentional blink? *Journal of Experimental Psychology. Human Perception and Performance*, *18*(3), 849–860. 10.1037/0096-1523.18.3.849 10.1037//0096-1523.18.3.849 1500880

[CR36] Raymond, J. E., Shapiro, K. L., & Arnell, K. M. (1995). Similarity determines the attentional blink. *Journal of Experimental Psychology: Human Perception and Performance, 21(3),* 653-662. 10.1037//0096-1523.21.3.653 7790839

[CR37] Shapiro KL (1994). The attentional blink: The brain's “eyeblink”. Current Directions in Psychological Science.

[CR38] Shapiro KL, Caldwell J, Sorensen RE (1997). Personal names and the attentional blink: A visual" cocktail party" effect. Journal of Experimental Psychology: Human Perception and Performance.

[CR39] Shapiro KL, Raymond JE, Arnell KM (1994). Attention to visual pattern information produces the attentional blink in rapid serial visual presentation. Journal of Experimental Psychology: Human Perception and Performance.

[CR40] Shapiro KL, Raymond JE, Arnell KM (1997). The attentional blink. Trends in Cognitive Sciences.

[CR41] Taatgen NA, Juvina I, Schipper M, Borst JP, Martens S (2009). Too much control can hurt: A threaded cognition model of the attentional blink. Cognitive Psychology.

[CR42] Tressoldi, P. E., & Giofrè, D. (2015). The pervasive avoidance of prospective statistical power: Major consequences and practical solutions. *Frontiers in Psychology, 6*, 726. 10.3389/fpsyg.2015.00726 10.3389/fpsyg.2015.00726 PMC4446541 26074863

[CR43] van de Schoot, R., Kaplan, D., Denissen, J., Asendorpf, J. B., Neyer, F. J., & van Aken, M. A. G. (2014). A gentle introduction to Bayesian analysis: applications to developmental research. *Child Development, 85*, 842-860. 10.1111/cdev.12169 10.1111/cdev.12169 PMC4158865 24116396

[CR44] Wagenmakers, E.-J., Verhagen, J., Ly, A., Bakker, M., Lee, M. D., Matzke, D., … Morey, R. D. (2014). A power fallacy. *Behavior Research Methods, 47*(4), 913-917. 10.3758/s13428-014-0517-4 10.3758/s13428-014-0517-4 25271090

[CR45] Watanabe S (2010). Asymptotic equivalence of Bayes cross validation and widely applicable information criterion in singular learning theory. Journal of Machine Learning Research.

[CR46] Weichselgartner E, Sperling G (1987). Dynamics of automatic and controlled visual attention. Science.

[CR47] Wicherts, J. M., & Bakker, M. (2012). Publish (your data) or (let the data) perish! Why not publish your data too? *Intelligence, 40*(2), 73–76. 10.1016/j.intell.2012.01.004

[CR48] Wyble B, Bowman H, Nieuwenstein M (2009). The attentional blink provides episodic distinctiveness: sparing at a cost. Journal of Experimental Psychology: Human Perception and Performance.

[CR49] Zwaan, R. A., Etz, A., Lucas, R. E., & Donnellan, M. B. (2018). Making replication mainstream. *Behavioral and Brain Sciences*, 1–61. 10.1017/S0140525X17001972 10.1017/S0140525X17001972 29065933

