# Supplementary material for: Two replications of Raymond, Shapiro, and Arnell (1992), The Attentional Blink
Source: Behav Res Methods. 2020 Aug 12;53(2):656–68. doi: 10.3758/s13428-020-01457-6 (PMC8062350; doi:10.3758/s13428-020-01457-6)
Supplement: Supplementary file 1 — (DOCX 3752 kb) [file 13428_2020_1457_MOESM1_ESM.docx]

# Supplemental Material for:

# Two replications of Raymond, Shapiro, and Arnell (1992), The Attentional Blink

# PART 1 – Posterior distributions from Experiments 1 and 2

Model fitting is described in the paper. The following material presents the posterior distributions of the fixed effects of the models that include the Condition (2 levels, within-participants: “control,” “experimental”; the baseline is “control”) x Lag (8 levels, within-participants: 1 to 8; the baseline is Lag 1) interaction. The models use the informative priors derived from Raymond et al. (1992). Table S1 reports the mean values and standard deviations of the posterior distributions for the Italian (Experiment 1) and the UK (Experiment 2) samples. Figure S1 shows the posterior distributions and the mean values of the prior distributions.

Table S1

*Means and SDs of the posterior distributions for the fixed effects (specifically, the Condition x Lag interaction) of the mixed-effects logistic models in the two experiments.*

| Model coefficient | Experiment 1:  Italian sample  (N = 98) |  | Experiment 2:  UK sample  (N = 29) |
| --- | --- | --- | --- |
|  | M (SD) |  | M (SD) |
| Intercept | 1.40 (.12) |  | 1.64 (.18) |
| Lag 2 | -.06 (.10) |  | -.31 (.19) |
| Lag 3 | -.14 (.10) |  | -.22 (.19) |
| Lag 4 | -.08 (.10) |  | -.41 (.19) |
| Lag 5 | -.11 (.10) |  | -.39 (.19) |
| Lag 6 | -.09 (.10) |  | -.22 (.19) |
| Lag 7 | -.19 (.10) |  | -.10 (.20) |
| Lag 8 | -.23 (.10) |  | -.34 (.19) |
| Experimental | -1.22 (.09) |  | -1.61 (.18) |
| Lag 2: Experimental | -.95 (.14) |  | -.73 (.27) |
| Lag 3: Experimental | -.86 (.14) |  | -.73 (.27) |
| Lag 4: Experimental | -.41 (.14) |  | .05 (.26) |
| Lag 5: Experimental | .26 (.14) |  | .57 (.26) |
| Lag 6: Experimental | .79 (.14) |  | .77 (.27) |
| Lag 7: Experimental | 1.04 (.14) |  | 1.07 (.27) |
| Lag 8: Experimental | 1.07 (.14) |  | 1.07 (.27) |


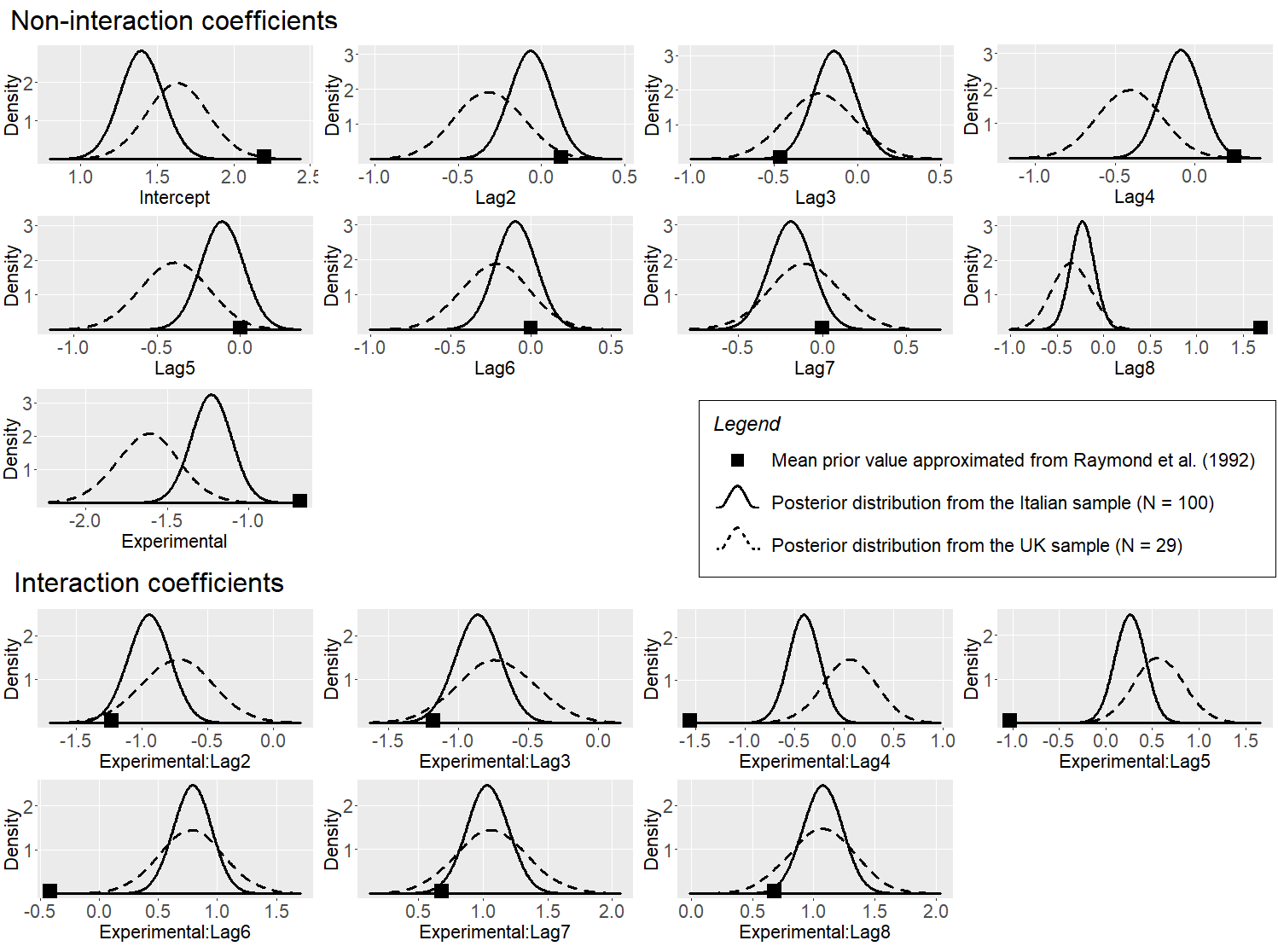


Figure S1. Posterior distributions of the parameters for Experiment 1 (Italian sample, N = 98) and Experiment 2 (UK sample, N = 29) are reported using the solid and dashed lines respectively. The square points represent the mean values of the prior distributions (derived from Raymond et al., 1992). As can be seen, for most parameters both posterior distributions exclude the mean prior value from their highest density area, and they differ from it in the same direction.

# PART 2 – Additional measures

## d’ scores

Table S2A

Mean (and standard deviation) of the d’ index for the probe detection task as a function of the experiment, condition and lag. The d’ was calculated with hit (response “yes” where the probe was actually presented) and false alarm (response “yes” where the probe was not presented) proportions. For the Raymond et al. (1992) study, the standard deviation could not be calculated as no measures of individual variability were reported; in this case, the hit rates were derived from the graph of the AB effect in their experiment 2, whereas the false alarm rates in the two conditions were reported in the text.

| Lag | Raymond et al. (1992) | | Experiment 1 | | Experiment 2 | |
| --- | --- | --- | --- | --- | --- | --- |
|  | Control | Experimental | Control | Experimental | Control | Experimental |
| 0 | 3.03 | 3.79 | 2.54 (.81) | 2.33 (.88) | 2.68 (.57) | 2.68 (.76) |
| 1 | 2.43 | 2.13 | 2.33 (.73) | 1.23 (.61) | 2.42 (.52) | 1.28 (.47) |
| 2 | 2.49 | 1.46 | 2.17 (.63) | .77 (.58) | 2.10 (.62) | .87 (.59) |
| 3 | 2.18 | 1.14 | 2.15 (.68) | .77 (.58) | 2.19 (.55) | .85 (.66) |
| 4 | 2.55 | 1.34 | 2.14 (.66) | 1.05 (.63) | 2.03 (.54) | 1.18 (.65) |
| 5 | 2.43 | 1.52 | 2.15 (.72) | 1.28 (.70) | 2.07 (.65) | 1.40 (.64) |
| 6 | 2.43 | 1.89 | 2.16 (.74) | 1.51 (.75) | 2.13 (.61) | 1.48 (.69) |
| 7 | 2.43 | 2.49 | 2.12 (.78) | 1.53 (.73) | 2.19 (.53) | 1.74 (.85) |
| 8 | 3.20 | 3.26 | 2.04 (.70) | 1.62 (.69) | 2.09 (.63) | 1.61 (.64) |

## Descriptive statistics for Experiment 1 divided by refresh rate (60 Hz vs. 70 Hz)

Table S2B

Descriptive statistics – means (and standard deviations) – of the proportions of correct probe detection in Experiment 1 as a function of Lag and Condition, divided by the refresh rate group.

| **Lag** | **Group: 60 Hz** | |  | **Group: 70 Hz** | |
| --- | --- | --- | --- | --- | --- |
|  | **Control** | **Experimental** |  | **Control** | **Experimental** |
| Lag 0 | .85 (.23) | .99 (.03) |  | .87 (.14) | 1.00 (.02) |
| Lag 1 | .80 (.21) | .52 (.33) |  | .79 (.22) | .50 (.30) |
| Lag 2 | .73 (.21) | .31 (.27) |  | .78 (.20) | .35 (.26) |
| Lag 3 | .74 (.21) | .36 (.25) |  | .75 (.22) | .30 (.26) |
| Lag 4 | .76 (.20) | .49 (.28) |  | .73 (.20) | .36 (.26) |
| Lag 5 | .74 (.22) | .63 (.23) |  | .74 (.22) | .50 (.28) |
| Lag 6 | .78 (.23) | .73 (.24) |  | .71 (.21) | .60 (.28) |
| Lag 7 | .73 (.26) | .74 (.26) |  | .74 (.21) | .67 (.24) |
| Lag 8 | .74 (.25) | 72 (.28) |  | .69 (.21) | .68 (.26) |

# PART 3 – Parameterizing the attentional blink (Cousineau et al., 2006)

## Bayesian estimation of parameters

We followed the parametrization of the attentional blink curve suggested by Cousineau, Charbonneau, and Jolicoeur (2006) and conducted an analysis of the attentional blink based on the four parameters that characterize the blink curve: 1) lag-1 sparing, 2) width, 3) amplitude, and 4) minimum of the blink (see Cousineau et al., 2006, Figure 1). Interestingly, the amplitude parameter estimated with the method suggested by Cousineau et al. (2006) seems coherent with the subsequent recommendation by MacLean and Arnell (2012) on how to estimate the magnitude of the attentional blink: the percentage of the vertical drop between the asymptotic (final) performance in the experimental condition and the minimum in the experimental condition. We also parametrized the control condition by fitting a linear regression to obtain 1) slope and 2) intercept of the control performance across lags. We used the Matlab functions provided by Cousineau and colleagues (2006) to obtain all parameters of the blink curve. In both fittings (i.e., experimental and control) we excluded the data gathered in the lag-0 condition (i.e., when the probe letter “X” is also the white target of the stream). The parameterization was fitted on the estimates obtained from the Bayesian models presented in the manuscript for Experiments 1 and 2. This allowed us to obtain not only the point estimates of the parameters, but also their 95% Bayesian credible intervals (BCI).

Regarding Experiment 1, the posterior distributions of width, lag-1 sparing, minimum, and amplitude indicated that width was replicated with precision: original study, width = .81; current study, width = .82, 95% BCI (.71, .95). Amplitude (a measure of the magnitude of the phenomenon calculated as the percent drop in accuracy between the asymptotic long-lags and the minimum performance at short-lags), was also similar, but slightly smaller in the original study: original study, amplitude = .52; current study, amplitude = .62, 95% BCI (56, .68). In contrast, lag-1 sparing and minimum were rather different: original study, lag-1 sparing = .34, current study, lag-1 sparing = .46, 95% BCI (.42, .51); original study, minimum = .48; current study, minimum = .28, 95% BCI (.23, .34).

Regarding Experiment 2, the parameters were remarkably similar to those estimated for Experiment 1. Therefore, they differed in the exact same way from the original estimates derived from Raymond et al. (1992): Width = .77, 95% BCI (.59, 1.00); lag-1 sparing = .46, 95% BCI (.38, .55); minimum = .24, 95% BCI (.17, .32); amplitude = .60, 95% BCI (.50, .70), slope = .00, 95% BCI (-.01, .01); intercept = .81, 95% BCI (.74, .86).

Figure S2 below shows the posterior distributions of the six parameters (four for the experimental condition, two for the control condition) estimated from the Bayesian regression models described in the article, separately for each experiment (Experiment 1: ITA; Experiment 2: UK), together with the same parameters derived from the original study (Figure 3A in Raymond et al., 1992). As can be seen, for most parameters both posterior distributions exclude the mean prior value from their highest density area, and they differ from it in the same direction.


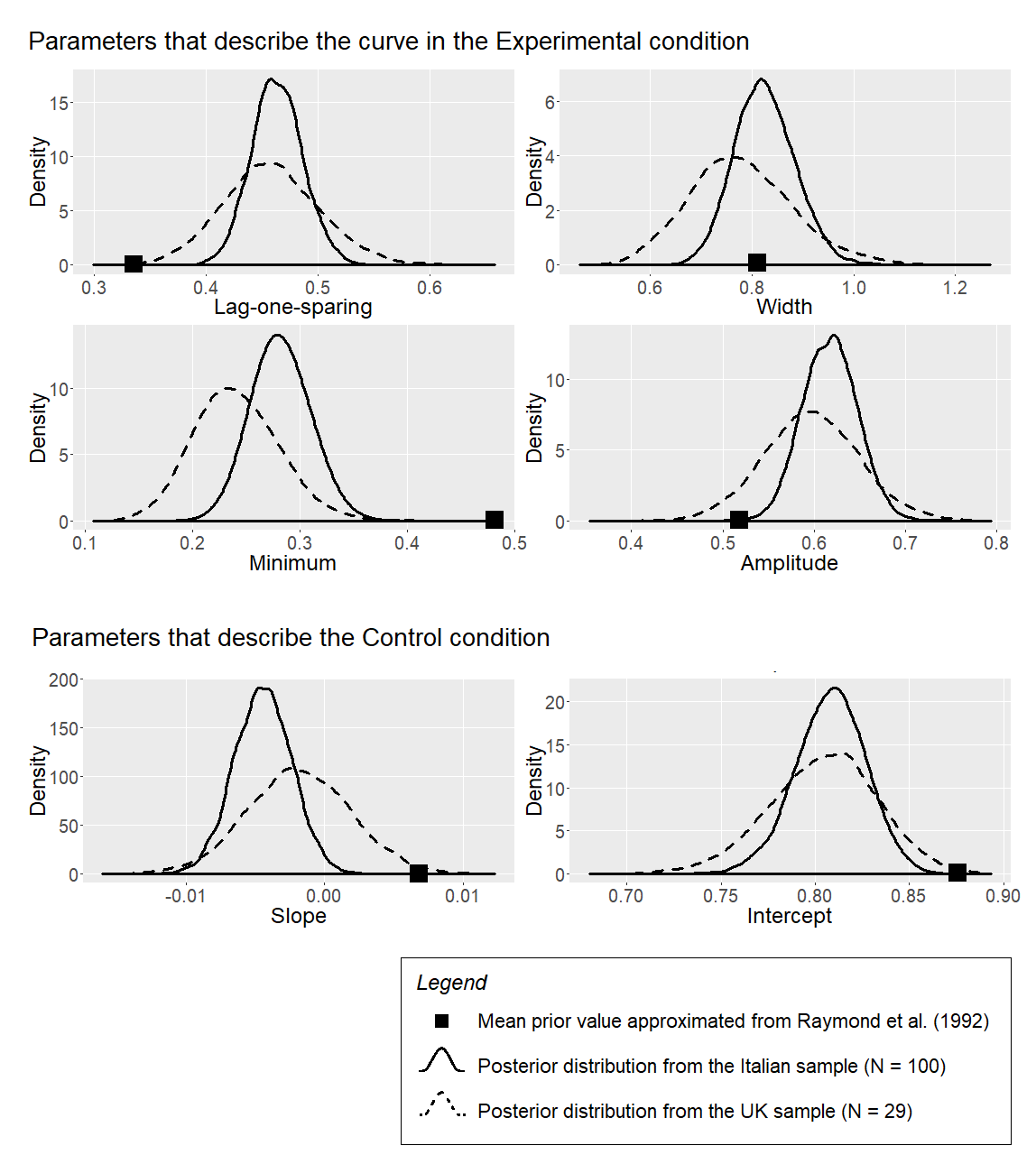


Figure S2. Posterior distributions of the parameters suggested by Cousineau and colleagues (2006), for Experiment 1 (Italian sample, N = 98) and Experiment 2 (UK sample, N = 29), reported using the solid and dashed lines respectively. The square points represent the values derived from the prior distributions (calculated from Figure 3A in Raymond et al., 1992).

## Did the original experiment differ from our replica due to a small sample size? A sampling procedure with N = 10

We also assessed whether the differences between the original study and our results could be explained by the small number of participants in the original study which may have led to inaccurate original estimates (see Morey & Lakens, 2016). The original experiment (Raymond et al., 1992) was run with 10 participants, whereas our replications were run on 98 participants in Padova-Italy (50 participants with SOA 100 ms and 48 participants with SOA 86 ms) and 29 participants in Liverpool-UK (SOA: 100 ms). Therefore, it is possible that the original results deviated from our estimated parameters due to the small sample of participants. For example, the overall high accuracy observed in the original experiment could be explained by a sample of participants that were, incidentally, good at the tasks.

For this analysis, we adopted a sampling procedure to define how likely were the original results under the hypothesis that the population from which the original participants had been sampled had identical parameters to those we observed in Experiment 1 and 2 — using the parameterization suggested by Cousineau et al., (2006) (i.e., width, lag-1 sparing, minimum, amplitude, slope and intercept).

A sampling procedure was conducted on our data. We resampled our data 10,0 times with N = 10 separately for three groups: Padova (SOA 100 ms, 60 Hz refresh rate), Padova (SOA 86 ms, 70 Hz refresh rate), Liverpool (SOA 100 ms, 60 Hz refresh rate). At each iteration, the data of the ten sampled participants were averaged separately by lag for the control condition and the experimental condition to obtain (i) the attentional blink curve, (ii) the corresponding performance in the control condition.

In the figures below (see Figures S3A and S3B), each graph represents the distribution of the parameters for the experimental condition and for the control condition when N = 10. In all figures, the vertical black dashed line represents the corresponding parameter estimated from Figure 3A of Raymond et al. (1992).


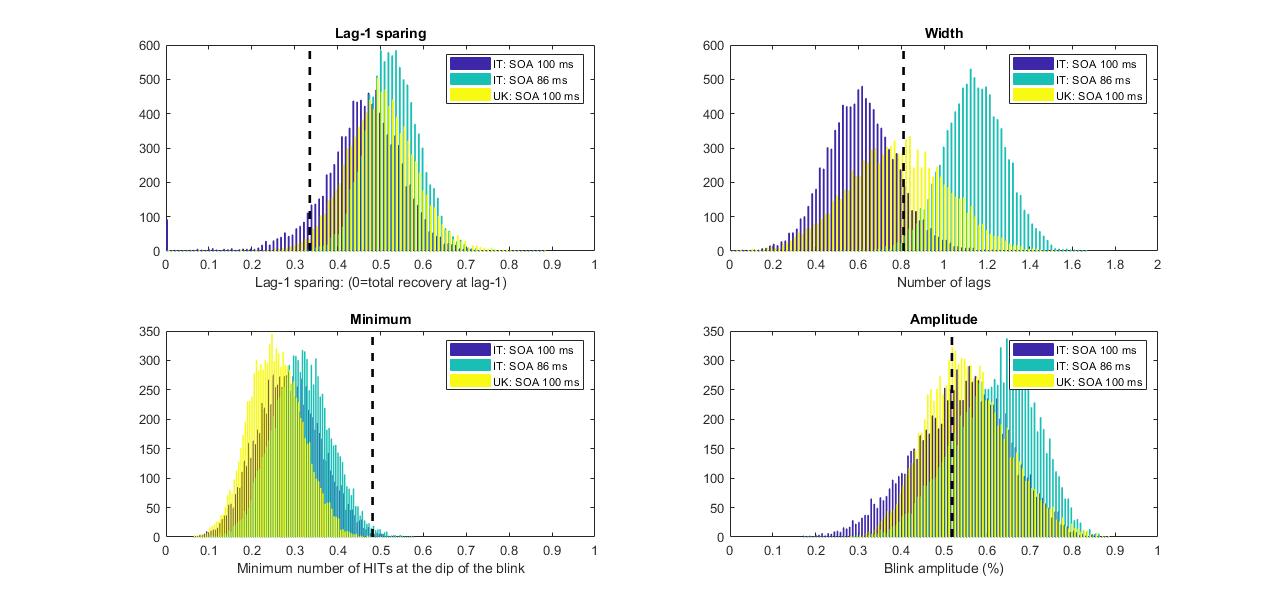


Figure S3A. Distribution of the parameters for the experimental condition. From top left, to bottom right: lag-1 sparing, width, minimum and amplitude. In each graph, the blue curve represents the results collected in Padova (60 Hz refresh rate), the turquoise curve represents the results collected in Padova (70 Hz refresh rate), and the yellow curve represents the results collected in Liverpool (60 hz refresh rate). The black vertical dashed line represents the corresponding parameter estimated from Figure 3A by Raymond et al. (1992) that used a SOA of 91 ms.


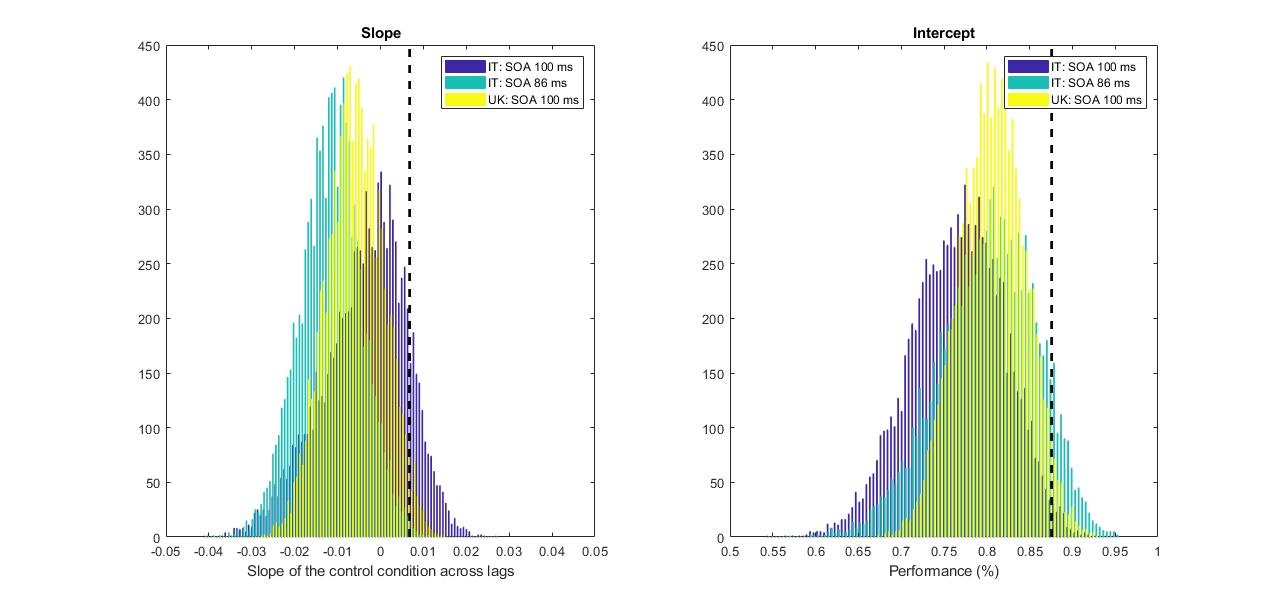


Figure S3B. Distribution of the parameters for the control condition. Left graph, slope of the linear fitting. Right graph, intercept of the linear fitting. In each graph, the blue curve represents the results collected in Padova (60 Hz refresh rate), the turquoise curve represents the results collected in Padova (70 Hz refresh rate), and the yellow curve represents the results collected in Liverpool (60 Hz refresh rate). The black vertical dashed line represents the corresponding parameter estimated from Figure 3A by Raymond et al. (1992) that used a SOA of 91 ms.

The Matlab functions and the data used to calculate this analysis can be found here: <https://osf.io/hp9nk/>

Regarding the experimental condition, the results of the Liverpool sample (60 Hz refresh rate) and the Padova sample (60 Hz refresh rate) overlap largely for all parameters. In contrast, the refresh rate seems to enlarge the width of the blink for the Padova sample (70 Hz refresh rate) in comparison to the other samples. The comparison of the Padova sample (70 Hz) and the other two samples does not reveal other substantial differences. The results observed by Raymond et al. (1992) fall within the distributions for width and amplitude. In contrast, they fall at the lower bound of the distribution (i.e., exceptional result) for lag-1 sparing and almost out of the distribution (i.e., very exceptional results) for the minimum parameter. Regarding the control condition, the results of all samples show a negligible negative slope close to zero. Also, Raymond et al. (1992) observed a similar result although the slope is positive. As far as the intercept (i.e., the average performance in the “control condition” across lags) is concerned, all our samples seem to overlap. However, the results gathered by Raymond et al. (1992) exhibit superior performance as compared to the large majority of those observed by sampling our data.

Finally, Table S3 reports the quantile of each parameter calculated from the data of Raymond et al. (1992) in comparison to our distributions (combining our two experiments). When the quantiles are extreme (i.e., close to 0 or to 1), that means that the original result largely deviates from our distributions, and thus that there was a small probability that the original results could be observed in a sample with N = 10 if the original study had sampled participants from the same population as we did (or, in other words, that it is highly likely that there was a difference in the population from which the participants were sampled).

In brief, the results of Raymond et al. (1992) seemed exceptional for lag-1 sparing (quantile .025) and minimum (quantile .995) of the attentional blink curve, whereas they were comparable with those we observed in the current experiment in terms of width (quantile .466) and amplitude (quantile .365). As far as the control condition is concerned, these results also seemed rather unlikely; the intercept of the linear fitting (i.e., the Hit rate of the participant in the control condition) was in quantile .960, and the slope was in quantile .965.

Table S3

Quantiles of the parameters estimated from Raymond et al. (1992) with reference to our sampling distributions with N = 10 (Experiment 1 and Experiment 2 combined).

| Parameter | Experimental Condition | Control condition |
| --- | --- | --- |
| Lag-1 sparing | .025 |  |
| Width | .466 |  |
| Minimum | .995 |  |
| Amplitude | .365 |  |
| Slope |  | .965 |
| Intercept |  | .960 |

Finally, we created a GIF animated picture which visually provides a series of examples of the comparisons across samples of identical size (i.e., N=10). In the GIF, the top left graph shows the comparison between samples (N=10) of Experiment 1 with the data of Raymond et al. (1992); the top right graph shows the comparison between samples (N=10) of Experiment 2 with the data of Raymond et al. (1992); the bottom left graph shows the comparison between samples (N=10) of Experiment 2 with the data of Experiment 1; the bottom right graph shows the comparison between samples (N=10) of Experiment 1 with the data of Experiment 2. Below we represent a one-frame example of the animated GIF. The GIF, the data and the script to generate a new GIF can be found here: <https://osf.io/hp9nk/>


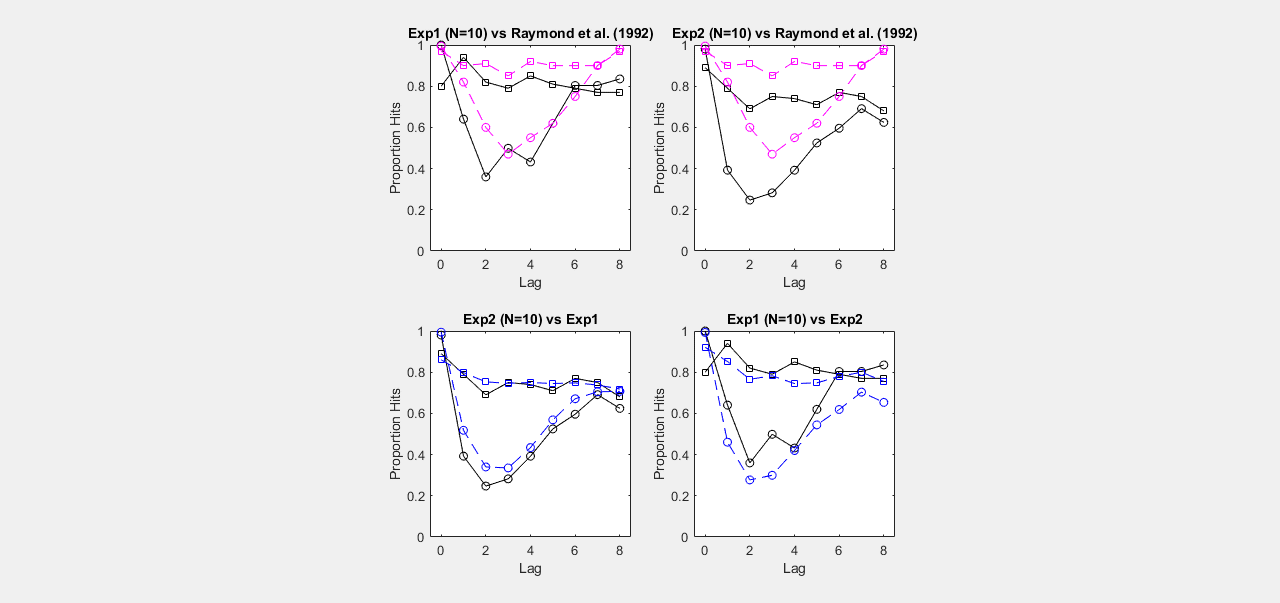


Figure S3C. One frame of the GIF animated picture that provides a series of examples of the comparisons across samples of identical size (i.e., N=10). The animated GIF, the data and the script to generate a new GIF can be found here: <https://osf.io/hp9nk/>

# PART 4 – Comparison of the font represented in Figure 1 of Raymond et al. (1992) and in the current replication

The following figure shows a comparison between the font used in the original study and the same sequence of characters used in the two replication studies that we conducted.


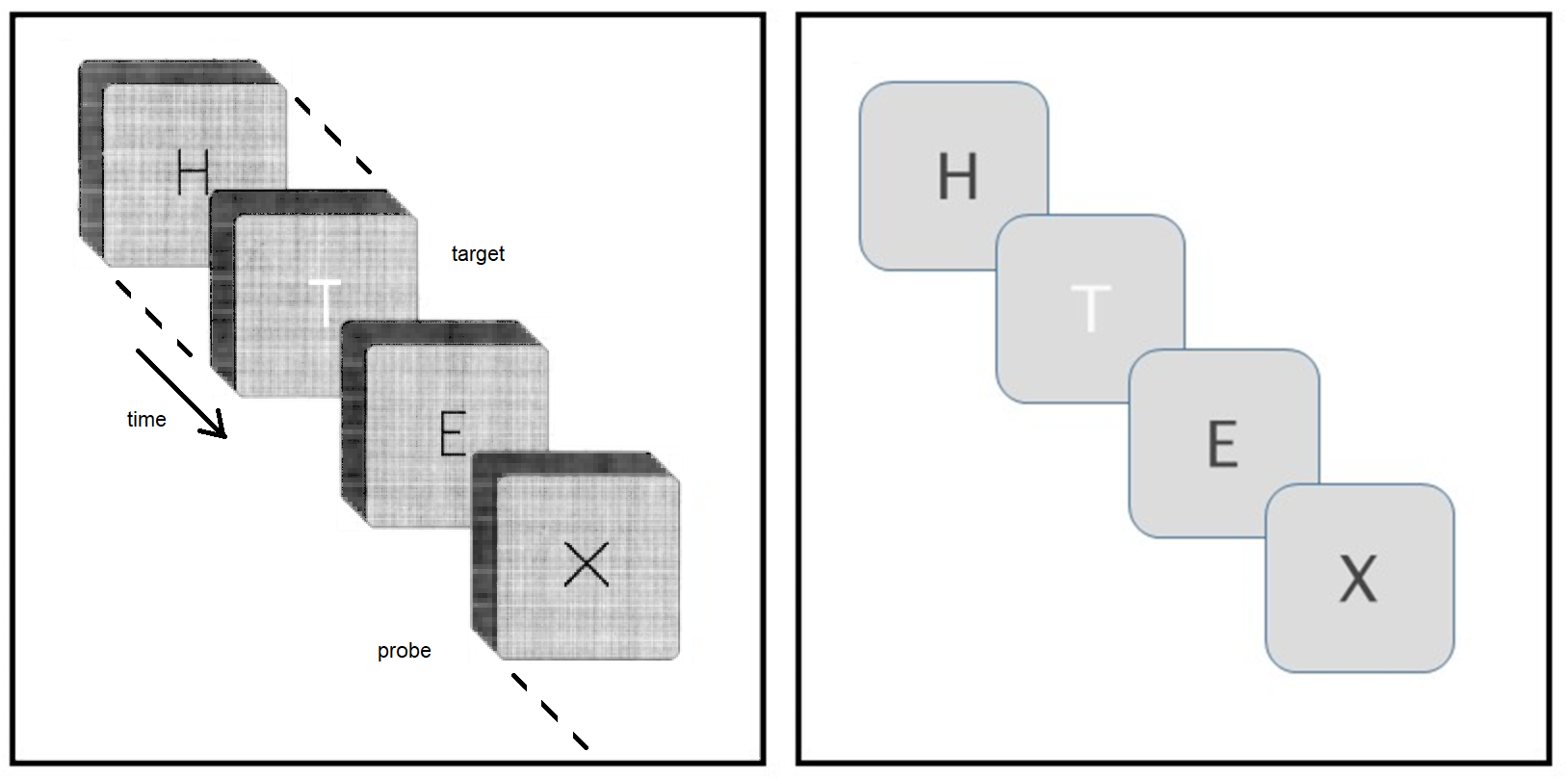


Figure S4. Comparison between the font used in the original study (left panel, adapted from Raymond et al., 1992) and the font used in our replication experiments (right panel).

# PART 5 – Sensitivity analysis: did the informed priors leverage the posterior distributions?

We reproduced the same Bayesian models presented in the manuscript for the condition x lag interaction but using uninformed default priors for all model parameters. Figure S5 below shows the posterior estimated accuracies in the probe identification as a function of condition and lag. As can be seen, the estimates are substantially indistinguishable from those presented in the Figure 1 of the manuscript for both experiments. Therefore, we did not investigate the parameters of the model as they would be identical to those already presented. The reasons why the prior distributions had substantially negligible effect on the posterior estimates can be summarized as follows:

- The dissimilarities between our data and the original study by Raymond and colleagues (1992) are limited. Most model parameters are thus practically the same. Therefore, the prior estimates (from Raymond et al., 1992) are largely in line with the likelihood of our data.
- Our Experiment 1 had a much larger sample size than the corresponding experiment by Raymond et al. (1992). Therefore, the prior is relatively weak compared with the data.
- The priors for Experiment 2 were set as even weaker. As explained in the manuscript, this was chosen after seeing some dissimilarities between our Experiment 1 and the original study, which raised doubts on the opportunity of using the same informed priors. Therefore, priors for Experiment 2 were even weaker, with larger SDs and very diffuse distributions.


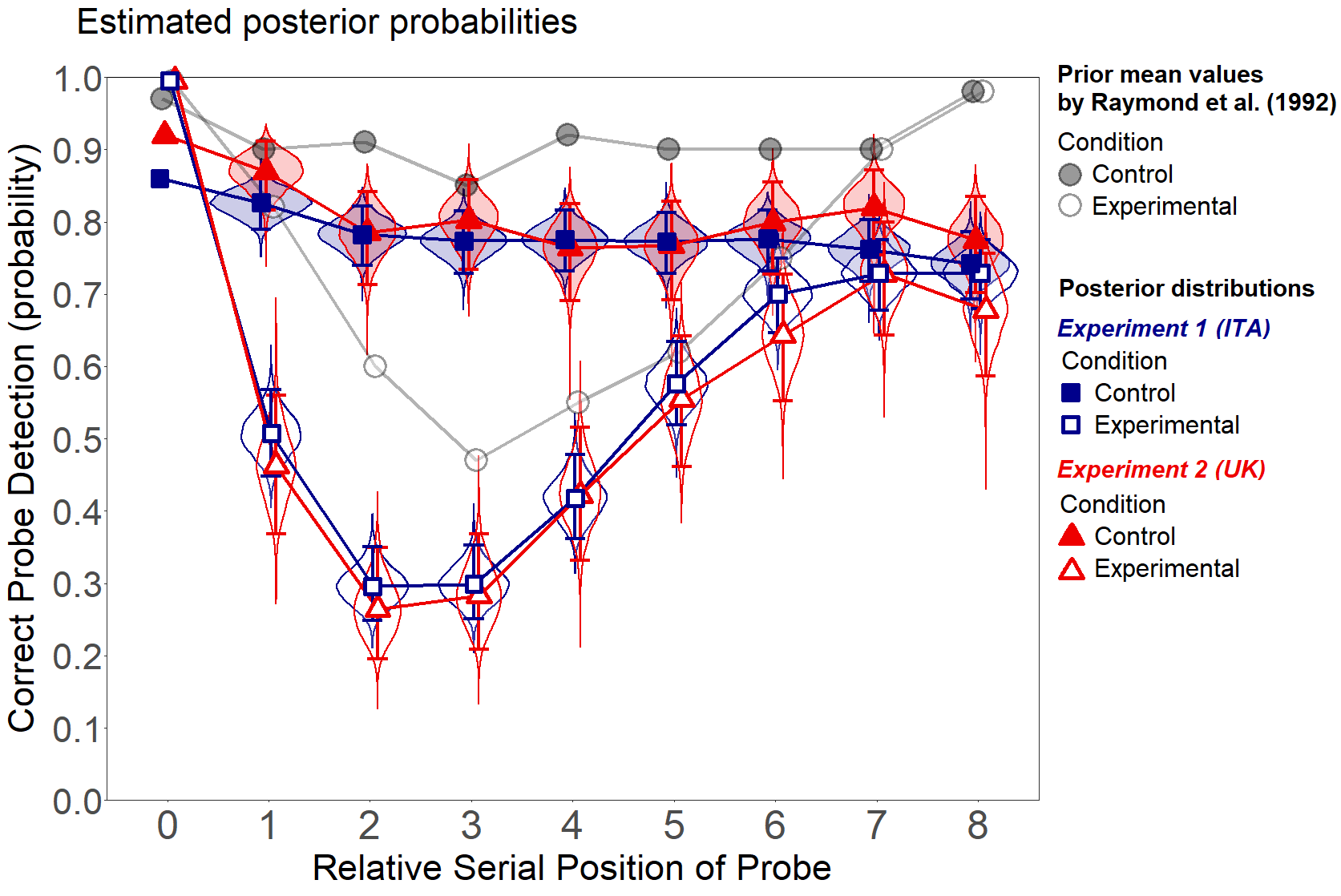


Figure S5. Estimated accuracy of correct probe detection (conditional to target identification) as a function of Condition and Lag, in Experiments 1 and 2, using uninformed default priors for all model parameters. Sensitivity analysis consists of a comparison with Figure 1 in the manuscript: as can be seen, the differences between the two figures are practically negligible. The error bars represent the 95% Bayesian credible intervals. The violins represent the entire posterior distributions. The circles represent the estimated prior mean values (thus reproducing the graph in the top panel of Fig. 3 in Raymond et al., 1992). Lag 0 was not included in the model, and its mean accuracies are shown for descriptive purposes.

# PART 6 – Comparisons between participants with High vs. Low accuracy to probe detection in the control condition

One could suspect that the differences between the results of the present study and those by Raymond et al. (1992) are bound to the difference in the probe detection task difficulty. In fact, in the original study the accuracy in the control condition was stable at around 90%, whereas in both our experiments it was about 80%. We conducted an additional analysis for our Experiment 1, in which we estimated the attentional blink separately in participants above the median (N = 44) and those at median or below (N = 54) in accuracy in the control condition. It could be expected that participants above the median show an attentional blink pattern that is nearly identical to the one reported by Raymond et al. (1992). Figure S6 below shows the estimated accuracy of correct probe detection as a function of condition and lag, in participants with high vs. low accuracy in the control condition.

The two groups have quite similar patterns of results. Unsurprisingly, the attentional blink is slightly larger in magnitude for the “high accuracy” group. This could be easily expected because those participants were selected for having high accuracy at the control condition, but not necessarily also in the experimental condition. One the one hand, the attentional blink pattern looked similar in the two groups in so far as the timing (reduced lag-one sparing, peak, and end of the blink) of both differed from the original results by Raymond et al. (1992). On the other hand, an overall visual inspection suggests that the pattern of the high accuracy group is similar to that observed by Raymond et al. (1992) in several aspects, including the majority of the point estimates. We cannot exclude that the difference in the timing of the blink we observed in our experiments in comparison to Raymond et al. (1992) emerged because of the difference in probe detection that was lower in our experiments in comparison to Raymond et al. (1992).


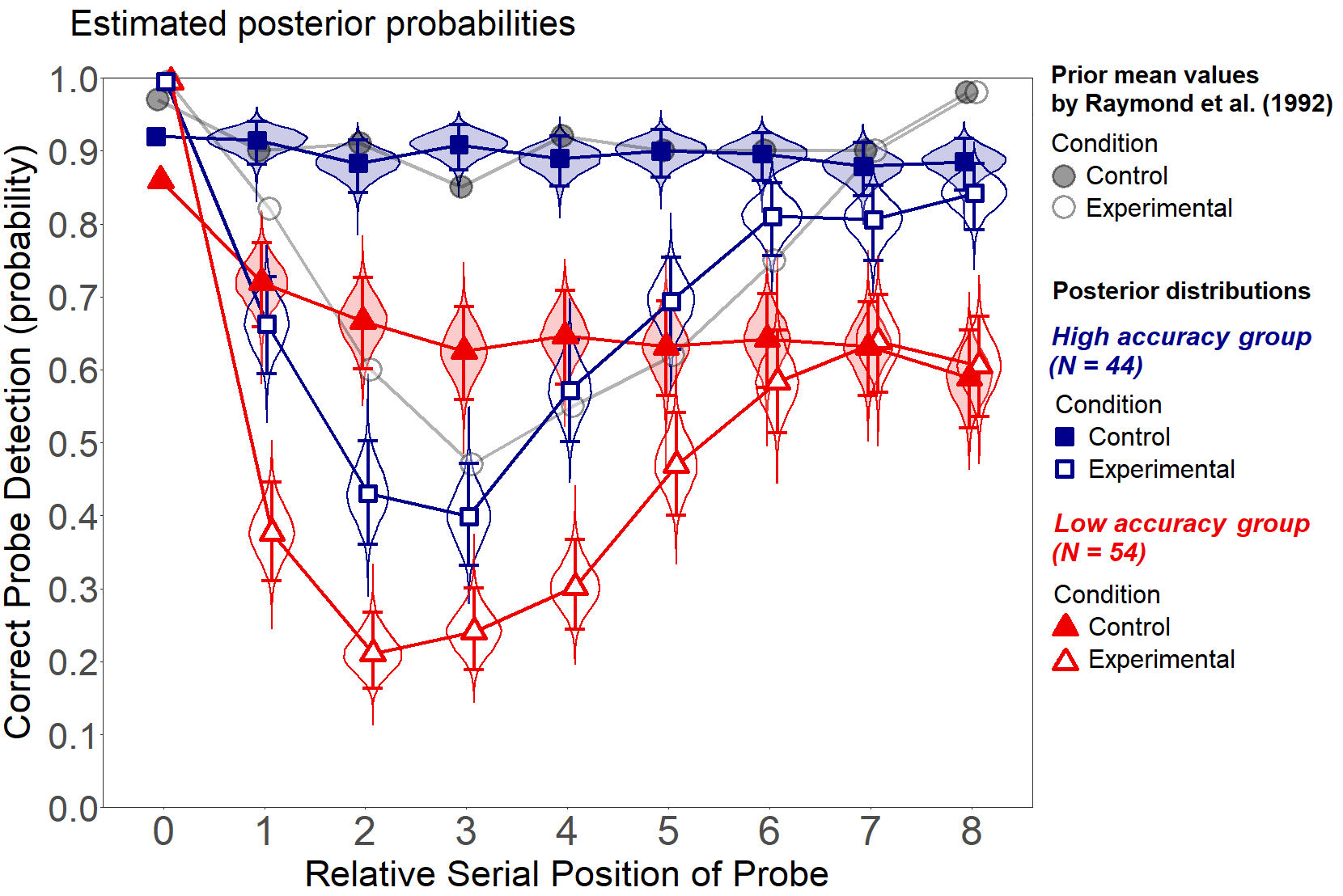


Figure S6. Estimated accuracy of correct probe detection (conditional to target identification) as a function of Condition and Lag. Data are presented separately for high accuracy participants (i.e., performance in control condition above the median) and low accuracy participants (i.e., performance in control condition below the median). Sensitivity analysis consists of a comparison with Figure 1 in the manuscript: as can be seen, the differences between the two figures are practically negligible. The error bars represent the 95% Bayesian credible intervals. The violins represent the entire posterior distributions. The circles represent the estimated prior mean values (thus reproducing the graph in the top panel of Fig. 3 in Raymond et al., 1992).

# PART 7 – Target-identification errors

In the paper by Raymond et al. (1992), the authors analyzed the errors made by participants when they identified the target letter in the experimental condition. Here we report a table of descriptive statistics and one graph that compares the results of the present experiments with those observed by Raymond et al. (1992). For such a comparison, the results of Raymond et al. (1992) were partly copied from the original paper (p854) and partly extrapolated by eye from Figure 3B.

Table S7 compares the descriptive statistics reported in Raymond et al. (1992, p854) with the equivalent descriptive statistics calculated for Experiment 1 and 2. The data reported in the table are calculated separately for probe absent trials and probe present trials. The latter are calculated, respectively, for probe in position lag 1 and in position lag 0 (i.e., the probe is the target).

Table S7

Target identification errors calculated in the present experiments and in Raymond et al. (1992).

|  | *Experiment 1* | *Experiment 2* | *Raymond et al. (1992)* |
| --- | --- | --- | --- |
| *Probe absent* | | | |
| *Errors* | *14.0%* | *22.2%* | *22%* |
| *Intrusion (i.e., +1) errors* | *6.1%* | *9.2%* | *11%* |
| *Intrusion errors over the total number of errors* | *43.6%* | *41.5%* | *50%* |
| *Intrusion (i.e., +2) errors* | *1.1%* | *1.7%* | *2%* |
| *Probe in position +1* | | | |
| *Errors* | *16.5%* | *33.7%* | *40%* |
| *Intrusion (i.e., +1), errors* | *8.2%* | *17.7%* | *33%* |
| *Intrusion errors over the total number of errors* | *49.4%* | *52.5%* | *82.5%* |
| *Probe in position 0* | | | |
| *Errors* | *13.4%* | *13.3%* | *10%* |
| *Intrusion errors over the total number of errors* | *46.6%* | *40%* | *80%* |


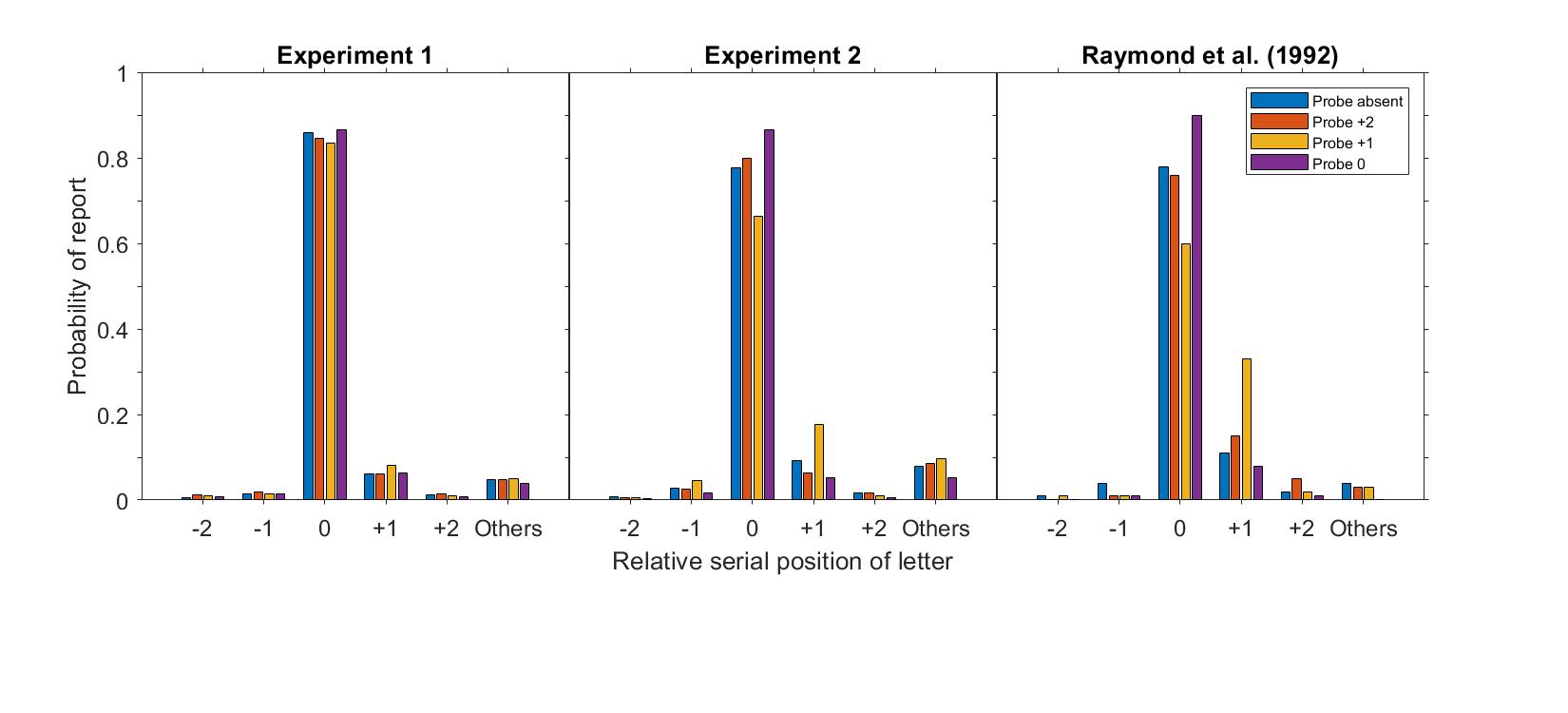


Figure S7. *Probability of reporting a letter as the target as a function of the relative serial position of the letter in the stream in comparison to the target. Negative values are positions before the target.*

# References (for the Supplemental material)

Cousineau, D., Charbonneau, D., & Jolicoeur, P. (2006). Parameterizing the attentional blink effect. *Canadian Journal of Experimental Psychology, 60(3),* 175-189. doi:10.1037/cjep2006017

MacLean, M. H., & Arnell, K. M. (2012). A conceptual and methodological framework for measuring and modulating the attentional blink. *Attention, Perception, & Psychophysics, 74(6),* 1080-1097.

Raymond, J. E., Shapiro, K. L., & Arnell, K. M. (1992). Temporary suppression of visual processing in an RSVP task: An attentional blink? *Journal of Experimental Psychology. Human Perception and Performance, 18(3),* 849-860. doi:10.1037/0096-1523.18.3.849
